# Visualizer cognitive style modulates the operation of object-based attention in processing bindings in visual working memory

**DOI:** 10.3389/fpsyg.2026.1875838

**Published:** 2026-10-12

**Authors:** Runze Li, Fengling Wei, Jukka Hyönä, Shouxin Li, Qian Zhang

**Affiliations:** 1 Faculty of Psychology, Shandong Normal University, Jinan, China; 2 Shandong Provincial Key Laboratory of Brain Science and Mental Health, Jinan, China; 3 Academic Affairs Office, Laiwu Vocational and Technical College, Jinan, China; 4 Department of Psychology and Speech-Language Pathology, University of Turku, Turku, Finland

**Keywords:** binding, event-related potentials, object-based attention, visual working memory, visualizer cognitive style

## Abstract

This study aims to investigate how visualizer cognitive style modulates the operation of object-based attention in processing bindings in visual working memory (VWM). Two behavioral experiments and one event-related potential (ERP) experiment were carried out. The revised Chinese version of the Object-Spatial Imagery Questionnaire was used to divide participants into object visualizers (OVs) and spatial visualizers (SVs). Participants maintained bindings in VWM alongside an interpolated task consuming object-based attention. The results of the behavioral experiments showed that, compared with SVs, OVs maintained bindings more stably but showed a larger cost when object-based attention was consumed. The ERP results showed that for OVs, when object-based attention was consumed, the P2 was reduced during VWM encoding, and the late positive component was enhanced during performance of the interpolated task. However, these modulations were absent for SVs. These findings suggest that visualizer cognitive style is associated with differences in binding stability, reliance on object-based attention, and resource engagement across VWM stages, advancing our understanding of how object-based attention contributes to the binding process in VWM.

## 1 Introduction

Visual information contains multiple features, such as colors, shapes, and directions. During perception, visual attention can bind multiple features into a unified binding, which is referred to as the binding process (Treisman, 1998; Treisman and Gelade, 1980). Both features and their bindings can be stored in visual working memory (VWM) (Luck and Vogel, 1997; Wheeler and Treisman, 2002), a limited-capacity system for temporarily storing and processing perceptual representations in the absence of sensory input (Baddeley, 2012; Hitch et al., 2025). Holding bindings in VWM requires the involvement of visual attention (Gao and Shen, 2024; Shen et al., 2015). However, exactly how visual attention contributes to processing bindings in VWM has not been determined.

The dual-task paradigm is commonly employed to examine attentional demands in VWM (Allen et al., 2006). This paradigm introduces an interpolated task that competes for attentional resources during the VWM maintenance stage to examine whether holding bindings in VWM is more affected by the interpolated task than holding features (Allen et al., 2006; Johnson et al., 2008; Shen et al., 2015). Visual attention is classified into space-based attention and object-based attention. Space-based attention refers to the process by which perceptual resources are devoted to a specific location to enhance the processing of all features at that location of the visual field, while object-based attention enhances the feature representations at the locations that define the selected object and can be allocated rapidly throughout the object (Carrasco, 2011; Cavanagh et al., 2023; Itti and Koch, 2001).

A series of studies has shown that object-based rather than space-based attention plays a special role in holding bindings in VWM (Fougnie and Marois, 2009; Gao et al., 2017; Johnson et al., 2008; Shen et al., 2015). For instance, Johnson et al. (2008) taxed space-based attention by inserting a visual-search task during VWM maintenance and found comparable interference with bindings and their constituent features. In contrast, Shen et al. (2015) used a feature-report task based on Duncan’s (1984) paradigm, in which participants selected one of two overlapping objects and reported its features, thereby taxing object-based attention. They found that bindings were selectively more impaired by the interpolated task than features, suggesting that object-based attention underlies the rehearsal of bindings in VWM. Furthermore, the role of object-based attention is robust across binding types, VWM activation states, and the number of features within the bindings, suggesting that object-based attention underlies a broad range of binding forms (Gao et al., 2017; He et al., 2020; Lu et al., 2019; Zhou et al., 2021). Researchers propose that the re-entry process in perception may also underlie the operation of object-based attention in maintaining bindings in VWM. The re-entry process involves feedback signals from the higher visual cortex (e.g., parietal lobe) to the early visual cortex, serving to verify the correctness of the bindings through bottom-up signals and to consolidate stable bound representations, with object-based attention operating through this re-entry process as a rehearsal mechanism during the binding process in VWM (Gao and Shen, 2024; Shen et al., 2015). However, it remains unclear how object-based attention operates in VWM.

Studies have found that the properties of binding representations modulate the degree to which object-based attention is required for bindings (Sun et al., 2025; Wan et al., 2020). Unlike holding bindings comprising separate features (e.g., color-shape bindings), holding integral bindings defined by tightly bound perceptual characteristics that cannot be processed independently (e.g., width and height of an object) does not require more object-based attention than holding features (Wan et al., 2020). Similarly, once bindings are consolidated into long-term memory (LTM), maintaining them does not require more object-based attention than feature maintenance (Sun et al., 2025). Researchers propose that the integral bindings and LTM representations are inherently stable and less easily disrupted by other information. According to the hypothesis of the re-entry process, object-based attention may not spread passively to encompass the target object but rather its allocation is a dynamic and active process (Spalek et al., 2024). Therefore, the stability of bindings may depend on how object-based attention is allocated across various stages of VWM.

The stability of VWM representations is modulated by the visualizer cognitive style (Li et al., 2011). The visualizer cognitive style, a typical cognitive style, characterizes two imagery abilities that individuals use to acquire and process visual information (Kozhevnikov et al., 2005). Using the Object–Spatial Imagery Questionnaire (OSIQ), researchers distinguish two types of visualizers: object visualizers (OVs) and spatial visualizers (SVs) (Blajenkova et al., 2006). OVs have advantages in processing images holistically, integrating them as a single perceptual unit, leading them to excel in constructing pictorial objects and outperform SVs in tasks requiring the generation of high-resolution images (e.g., recognizing degraded objects). In contrast, SVs have advantages in processing images analytically, part by part, using spatial relations to arrange and analyze the components. SVs outperform OVs in representing dynamic spatial relations and transformations (e.g., mental rotation) (Kozhevnikov et al., 2010, 2005). The differences in visualizer cognitive style reflect a trade-off between object and spatial visualization abilities within a limited capacity of visual attention (Kozhevnikov et al., 2010). With respect to the neural underpinnings, a functional magnetic resonance imaging study showed that despite equivalent behavioral performance during the object imagery task, OVs exhibited lower neural activity in the object-processing pathway than SVs, indicating greater neural efficiency for OVs than for SVs (Motes et al., 2008). Moreover, a study of VWM revealed that for object representations, OVs maintained stable performance with prolonged retention intervals, whereas SVs showed a decline. In that study, OVs exhibited higher positive prefrontal slow potentials than SVs, indicating that the superior stability in OVs stems from higher neural efficiency during the retention stage of VWM (Li et al., 2011).

Based on the prior evidence, the mechanism underlying the advantage of OVs over SVs in object VWM processing may explain how object-based attention plays a role in maintaining bindings in VWM. We hypothesized that visualizer cognitive style may modulate the stability of binding maintenance in VWM by affecting the relative reliance on object-based attention during the binding process. How do individuals employing different visualizer cognitive styles allocate resources across distinct stages of VWM to support stable bindings? We explored this question from the perspective of visualizer cognitive style by combining behavioral and ERP experiments, providing new evidence about the mechanisms by which object-based attention operates in VWM to maintain bindings stably. Three experiments were carried out.

To test whether the advantage of OVs over SVs extends to the feature bindings in VWM, in Experiment 1, we investigated whether OVs hold bindings in VWM more stably than SVs. A delayed matching task with a long or short delay interval was used to assess the stability of binding maintenance. We hypothesized that OVs and SVs would perform comparably in the short delay condition, while OVs would perform better than SVs in the long delay condition. In Experiment 2, we adopted a dual-task paradigm to investigate whether OVs and SVs differed in their dependence on object-based attention when retaining bindings. The hypothesis was that compared to SVs, OVs who excel in binding maintenance within VWM would exhibit greater impairment when object-based attention was consumed by an interpolated task. In Experiment 3, we used the ERP technique to investigate how the visualizer cognitive style modulates neural resource allocation across distinct VWM stages under concurrent demands on object-based attention. The P2 and late positive component (LPC) were used as ERP indicators of VWM encoding and interpolated task performance, respectively. P2 reflects the recruitment of attentional resources during VWM encoding (Smith, 1993; Wolach and Pratt, 2001). OVs may maintain bindings more stably because they could more dynamically allocate attention across distinct VWM stages according to the task demands, compared to SVs. Thus, the hypothesis was that OVs would elicit a smaller P2 during VWM encoding in the interpolated task-present condition than in the interpolated task-absent condition, while SVs would elicit comparable P2 amplitudes in the two conditions. In the dual-task paradigm, LPC has been linked to both VWM retention and the processing demands of the interpolated task (Gunseli et al., 2014a,b; Jongen and Jonkman, 2011; Pratt et al., 2011). Thus, the hypothesis was that OVs would elicit a larger LPC in holding bindings in VWM in the condition in which the interpolated task competes for object-based attention than in the condition without the interpolated task, while SVs would elicit comparable LPC amplitudes in the two conditions.

## 2 Experiment 1: holding bindings in VWM by OVs and SVs

### 2.1 Method

#### 2.1.1 Transparency and openness

The data of the reported experiments are available on the Open Science Framework (https://osf.io/f69rh/?view_only=a7d33b50165848bea8a562fe8f66e554, view-only link until acceptance of the corresponding manuscript). This study’s design and its analysis were not preregistered. We report below how we determined our sample size, all data exclusions, all manipulations, and all measures in the study.

#### 2.1.2 Revised version of the object-spatial imagery questionnaire

We employed the revised Chinese version of the Object-Spatial Imagery Questionnaire (OSIQ-R) by Li et al. (2011) to classify the participants as either OVs or SVs. It consists of 15 items in the object imagery scale (e.g., “When I read a novel, I can usually form a detailed and vivid image of the scene based on the description”) and 15 items in the spatial imagery scale (e.g., “I can easily imagine and mentally rotate 3-dimensional geometric figures”). Participants were instructed to rate each statement on a 5-point scale ranging from 1 (total disagreement) to 5 (total agreement). Object and spatial imagery scores were computed separately by summing the ratings of the 15 items for each scale. For the present study, the Cronbach’s α coefficients for the object and spatial scales were 0.78 and 0.73, indicating acceptable reliability.

#### 2.1.3 Participants

A total of 1,290 college students were initially tested with the OSIQ-R. OVs were defined as participants scoring in the top 30% on the object imagery scale and in the bottom 30% on the spatial imagery scale (Li et al., 2011). Conversely, SVs were defined as participants scoring in the bottom 30% on the object imagery scale and in the top 30% on the spatial imagery scale. Based on the OSIQ-R scores, 123 students [*M*_object-scores_ = 3.90, 95% CI (3.85, 3.96); *M*_spatial-score_ = 2.35, 95% CI (2.30, 2.39)] were classified as OVs and 83 students [*M*_object-scores_ = 2.86, 95% CI (2.80, 2.93); *M*_spatial-score_ = 3.42, 95% CI (3.36, 3.48)] were classified as SVs. These participants were then included in the object or spatial visualizer pools, from which subjects for the three experiments were randomly selected.

The sample size for the current experiment was calculated using G*Power (Version 3.1.9; Faul et al., 2009), with *F* tests: ANOVA, repeated measures, within–between interaction selected as the statistical test. The effect-size estimate was informed by Li et al. (2011), a study with a similar experimental design, from which an effect size of *f* = 0.45 was estimated. Thus, we adopted a large effect size of *f* = 0.40. The analysis used an alpha level (*α*) = 0.05, and the statistical power (1 − β) = 0.80, two groups, two repeated measurements, a correlation of 0.50 among repeated measures, and a non-sphericity correction of ϵ = 1.00. The analysis indicated a minimum sample size of 16 per group.

Twenty OVs [13 females, 18–22 years old; *M*_object-scores_ = 3.95, 95% CI (3.81, 4.10), *M*_spatial-score_ = 2.76, 95% CI (2.62, 2.90)] and 20 SVs [12 females, 18–20 years old; *M*_object-scores_ = 2.40, 95% CI (2.31, 2.49); *M*_spatial-score_ = 3.30, 95% CI (3.21, 3.39)] were randomly selected from the OV and SV pools for this experiment. The difference in gender distribution was not significant between the two groups (*χ*^2^ = 1.07, *p* = 0.744). All participants had normal or corrected-to-normal vision and normal color vision. Each participant was naive regarding the purpose of the study and participated in only one of the three experiments. All the participants were right-handed and had no history of neurological problems. The present study was conducted in accordance with the APA Ethical Principles and approved by the Ethics Committee of Faculty of Psychology, Shandong Normal University.

#### 2.1.4 Materials and apparatus

The stimuli were 36 objects (1.5° × 1.5°). Each was defined by a random combination of 6 shapes and 6 colors (red: 255, 0, 0; green: 0, 255, 0; blue: 0, 0, 255; yellow: 255, 255, 0; purple: 255, 0, 255; white: 255, 255, 255; see Figure 1). The memory array contained 3 items distributed horizontally in the center of the screen, with an inter-circle distance of no less than 2.5°. The probe was located 2.5° below the center of the screen.

**Figure 1:**
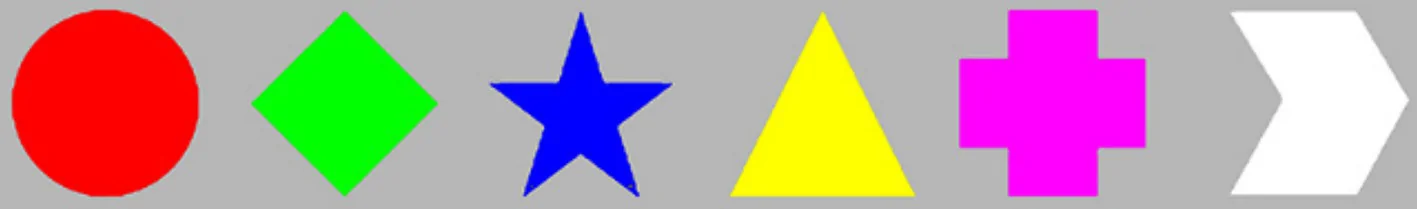
The stimuli used in Experiment 1.

The stimuli were displayed with E-prime 2.0 (Psychology Software Tools, Inc., Sharpsburg, Pennsylvania, USA) on a gray (RGB: 125, 125, 125) background on an LCD monitor with a resolution of 1,024 × 768 pixels at a 60 Hz refresh rate.

#### 2.1.5 Design and procedure

A 2 (cognitive style: *OVs* vs. *SVs*) × 2 (delay: *short* vs. *long*) mixed experimental design was utilized, in which cognitive style served as a between-subjects factor, and the delay served as a within-subjects factor. The delay was the retention interval between the disappearance of the memory array and the presentation of the test probe: the short delay condition was 1,000 ms, and the long delay condition was 3,000 ms. There were two blocks: a binding probe with a short delay and a binding probe with a long delay.

During the experiment, participants were seated in a quiet room, approximately 57 cm from the screen. As shown in Figure 2, each trial started with the presentation of two black digits for 1,000 ms. Participants were instructed to repeatedly articulate these two digits aloud throughout the entire trial to prevent verbal coding of the stimuli. After a 500 ms fixation on a cross in the center of the screen, the memory array was presented for 500 ms. Next, a retention interval of 1,000 or 3,000 ms (short or long delay) followed. A probe was then presented for 2000 ms in the center of the lower screen, during which the participants determined whether the color and shape combination of the probe was identical to that of any of the items presented in the memory array by pressing a response key (pressing “F” for a “yes” response or “J” for a “no” response). In 50% of the trials, the probe was one of the items in the memory array, while in the remaining trials the probe was a novel conjunction formed by recombining two features from the memory array that originally belonged to different items. Each block had 48 trials, resulting in a total of 96 trials. Participants completed 20 practice trials before each block. The order of the two blocks was counterbalanced between participants. A 5 min break was given between the blocks. The task lasted approximately 30 min.

**Figure 2:**
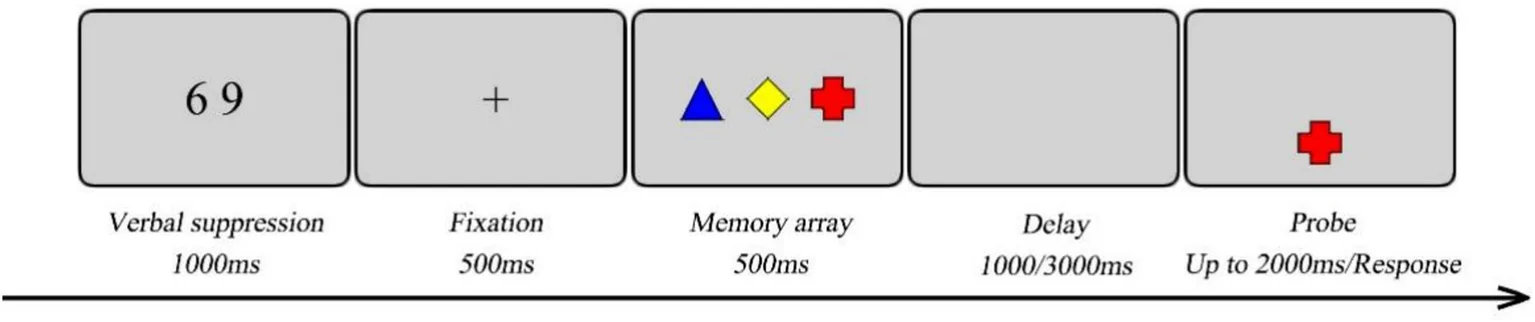
A schematic illustration of a trial in Experiment 1 (in this trial the probe was present in the memory array).

#### 2.1.6 Analysis

The statistical analysis was conducted with SPSS 26.0 (IBM SPSS Statistics, Armonk, NY: IBM Corp.). Accuracy was analyzed using a 2 (cognitive style) × 2 (delay) mixed repeated-measures analysis of variance (ANOVA). Significant interactions were followed up with simple-effects analyses.

### 2.2 Results

The two-way ANOVA on accuracy showed a significant main effect of cognitive style, *F* (1, 38) = 4.94, *p* = 0.032, η_p_^2^ = 0.12. OVs [*M* = 0.82, 95% CI (0.80, 0.85)] performed better than SVs [*M* = 0.78, 95% CI (0.76, 0.81)]. The main effect of delay was also significant, *F* (1, 38) = 38.45, *p* < 0.001, η_p_^2^ = 0.50. The accuracy in the short delay condition [*M* = 0.84, 95% CI (0.81, 0.86)] was better than that in the long delay condition [*M* = 0.77, 95% CI (0.75, 0.79)]. The interaction was significant as well, *F* (1, 38) = 13.91, *p* < 0.001, η_p_^2^ = 0.27. A simple-effects analysis showed that the accuracy for OVs was higher than that for SVs in the long delay condition, *F* (1, 38) = 16.82, *p* < 0.001, η_p_^2^ = 0.31, but not in the short delay condition, *F* (1, 38) = 0.023, *p* = 0.880, η_p_^2^ = 0.00 (see Figure 3).

**Figure 3:**
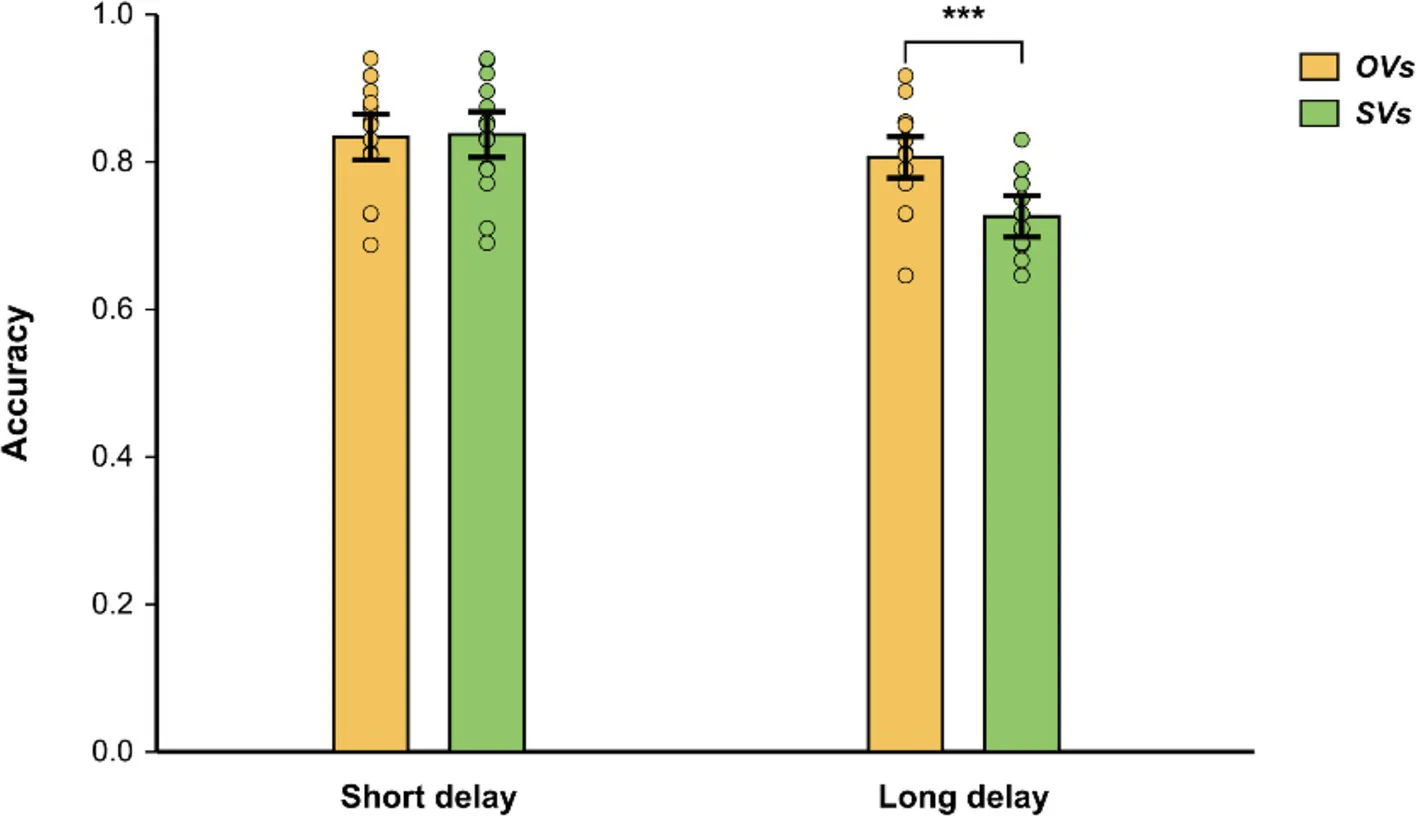
The mean performance accuracy for Experiment 1; each dot corresponds to the data of an individual participant. Bars represent the 95% CIs. OVs, object visualizers; SVs, spatial visualizers. ***p < 0.001.

### 2.3 Discussion

In Experiment 1, we investigated the color-shape binding performance in VWM of OVs and SVs. To manipulate the stability of VWM maintenance, we varied the retention interval between the memory array and the probe. Articulatory suppression was included to minimize the verbal maintenance of visual stimuli (Matsukura and Hollingworth, 2011). In addition, half of the probes were composed of recombined colors and shapes from the memory array, requiring participants to maintain their correct conjunctions, thereby explicitly engaging binding processes (Allen et al., 2006). The results showed that OVs and SVs performed comparably in the short delay condition, whereas OVs outperformed SVs in holding bindings in VWM in the long delay condition. That is, SVs showed a larger performance decrement in the long delay condition than OVs, which aligns with and extends the findings of Li et al. (2011) in revealing that OVs hold bindings in VWM more stably than SVs.

Prior studies have revealed that holding bindings in VWM requires more object-based attention than holding features (Gao et al., 2017; Shen et al., 2015; Zhou et al., 2021). Does the greater stability of bindings among OVs than among SVs result from OVs’ greater reliance on object-based attention in maintaining bindings in VWM? To examine this issue, we employed a dual-task paradigm in Experiment 2, in which an interpolated task was inserted during the VWM maintenance stage. The interpolated task requires participants to recognize a stimulus composed of two multi-feature objects and to report the feature of a target object by directing object-based attention to that object while ignoring the other (Duncan, 1984). This manipulation occupied object-based attention during VWM maintenance (Gao et al., 2017; Shen et al., 2015), which allowed a comparison of the role of object-based attention in binding between OVs and SVs. As a control, feature-based VWM was also examined to confirm that the effect was specific to binding.

## 3 Experiment 2: do OVs and SVs differ in their dependence on object-based attention in holding bindings in VWM?

### 3.1 Method

#### 3.1.1 Participants

The effect-size estimate was informed by Shen et al. (2015), a study with a similar experimental design, with an estimated effect size of *f* = 0.61. Thus, we adopted a large effect size of *f* = 0.40. The remaining parameters were the same as in Experiment 1, except for four repeated measurements. The estimated minimum sample size was 12 per group.

Twenty OVs [12 females, 18–20 years old; *M*_object-scores_ = 3.88, 95% CI (3.72, 4.04), *M*_spatial-score_ = 2.29, 95% CI (2.17, 2.42)] and 20 SVs [8 females, 18–20 years old; *M*_object-scores_ = 2.70, 95% CI (2.54, 2.86); *M*_spatial-score_ = 3.36, 95% CI (3.23, 3.48)] were randomly selected from the OV and SV pools for this experiment. The difference in gender distribution was not significant between the two groups (*χ*^2^ = 1.61, *p* = 0.206).

#### 3.1.2 Materials and apparatus

The binding stimuli were the same as those used in Experiment 1. For the feature stimuli, 6 shapes (filled with black: 0,0,0) and 6 vague, irregular, and noncanonically shaped color blobs were used (Shen et al., 2015; see Figure 4a).

**Figure 4:**
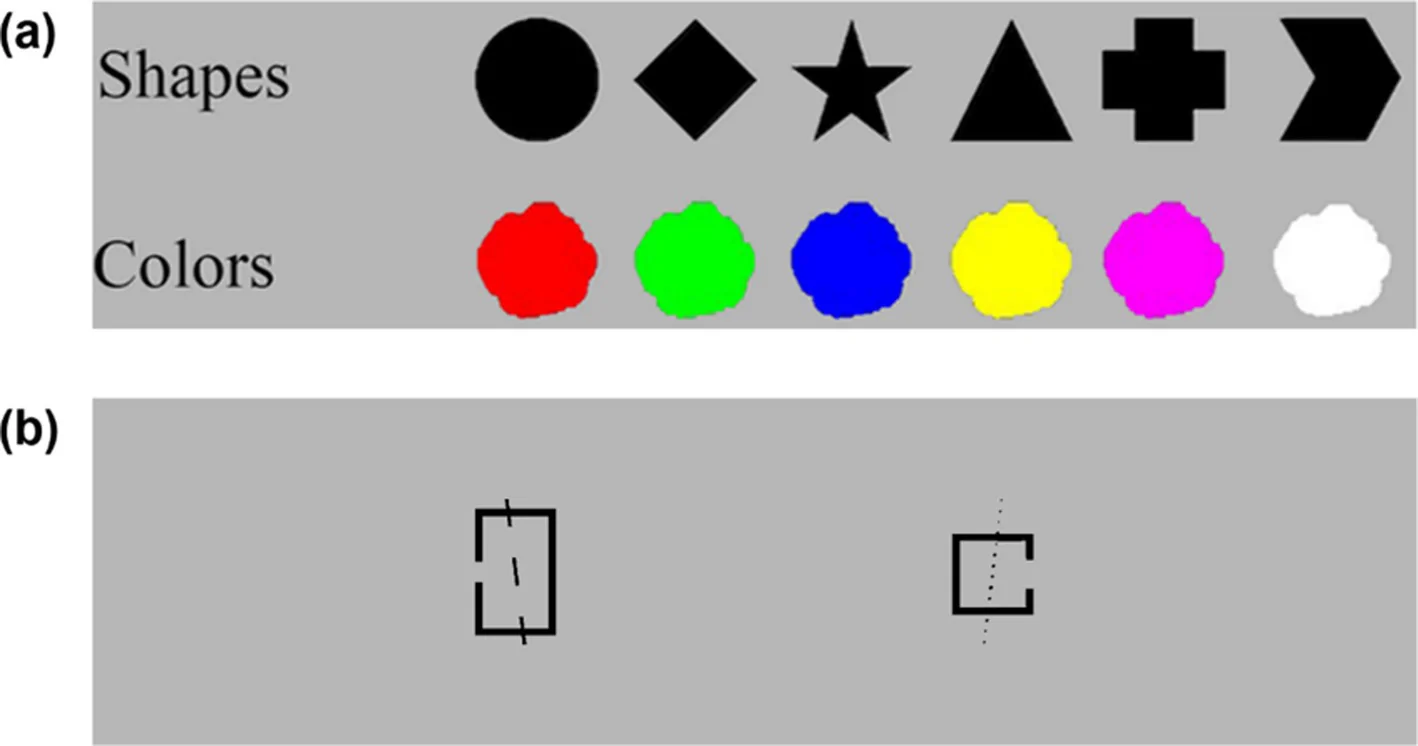
Example stimuli used in Experiment 2. **(a)** The shape and color stimuli used in Experiment 2. **(b)** Examples of the box-line stimuli used in the interpolated task. The left example shows a tall box with a left-side gap and a left-tilted dashed line, while the right example shows a short box with a right-side gap and a right-tilted dotted line.

The interpolated task was a feature report task (Shen et al., 2015; see Figure 4b). The box-line stimuli consisted of two superimposed objects: a box (0.67° wide) with a gap and a tilted line. The box was either short (0.67°) or tall (1.14°) and had a gap (0.20° wide) centered either on the left or right. The line (1.53° long) was dashed or dotted and tilted 8° to the left or right. The box-line stimulus was followed by a visual mask that was 1.62° in width and 2.10° in height. There were four feature report questions related to the box or the line: (1) box height (short vs. tall), (2) box gap orientation (left vs. right), (3) line type (dashed vs. dotted), and (4) line tilt (left vs. right).

The apparatus and software were the same as those used in Experiment 1.

#### 3.1.3 Design and procedure

A 2 (cognitive style: *OVs* vs. *SVs*) × 2 (interpolated task: *task-present* vs. *task-absent*) × 2 (probe: *feature* vs. *binding*) mixed experimental design was utilized, in which cognitive style served as a between-subjects factor and the interpolated task and the probe served as within-subjects factors. In the interpolated task, the participants were required to remember the box-line stimulus and answer two questions (see below). In the feature probe condition, the stimuli were shapes or colors, which were presented separately; in the binding probe condition, the stimuli were a combination of a color and a shape. Thus, there were 6 blocks: a color feature probe with the interpolated task present, a color feature probe with the interpolated task absent, a shape feature probe with the interpolated task present, a shape feature probe with the interpolated task absent, a binding probe with the interpolated task present, and a binding probe with the interpolated task absent.

During the experiment, participants were seated in a quiet room approximately 57 cm from the screen. The experimental procedure was in line with previous studies (Shen et al., 2015; see Figure 5). Each trial started with a fixation cross for 500 ms in the center of the screen. Then, the memory array was presented for 500 ms. Next, after a 500 ms delay, a box-line stimulus was presented for 200 ms, followed by a 200 ms visual mask. Two questions were then sequentially presented. In the task-present condition, participants were instructed to remember the box-line stimulus and report two box-line features by pressing “F” or “J” based on the presented alternatives. The first question was about the box, and the second was about the line (see above). In the task-absent condition, participants were instructed to ignore the questions by pressing the “spacebar” on the keyboard. The feature report question screen lasted a maximum of 2000 ms. Once participants made a response by pressing the corresponding key on the keyboard, the procedure immediately continued to the next step. After another 500 ms delay, a probe was presented for 2000 ms in the lower center of the screen. In the feature probe condition, participants were to determine whether the color or the shape matched any item in the memory array by pressing a response key (pressing “F” for a “yes” response or “J” for a “no” response). In 50% of the trials, the probe was one of the items in the memory array, while in the remaining trials, the probe was a new color or shape that did not appear in the memory array. In the binding conditions, the settings were identical to those in Experiment 1. Each block contained 48 trials resulting in a total of 288 trials. Participants completed 20 practice trials before each block. The order of the six blocks was counterbalanced between participants. A 5 min break was given between blocks. The task lasted approximately 70 min.

**Figure 5:**
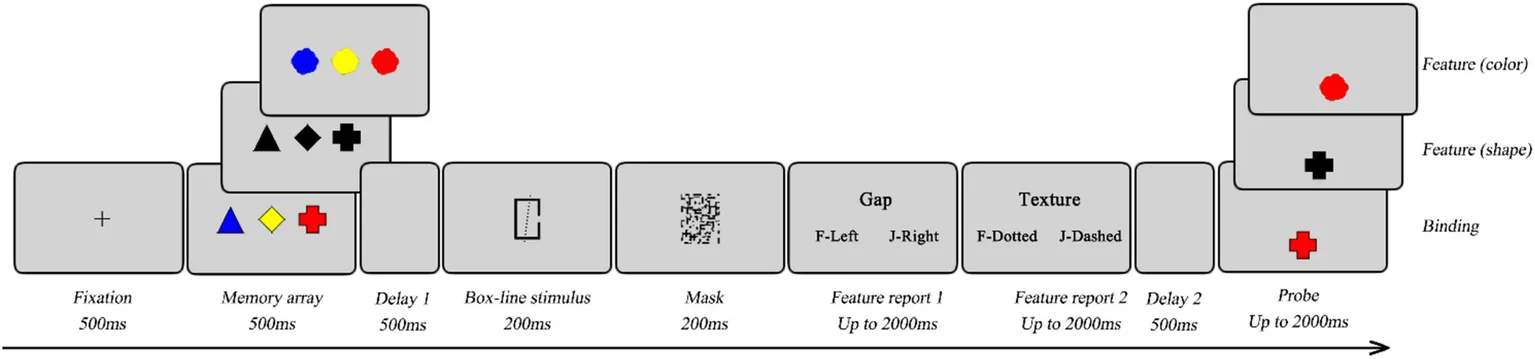
A schematic illustration of a trial in Experiment 2. The figure depicts the trials of the feature and the binding probe conditions, in which an interpolated task was used (in this trial the probe was present in the memory array).

#### 3.1.4 Analysis

Only trials with correct responses on the interpolated task were analyzed. To directly compare the feature and binding performance, the data for the color and shape conditions were collapsed for the analysis, with separate analyses presented in Appendix A. Performance accuracy was analyzed using a 2 (cognitive style) × 2 (interpolated task) × 2 (probe type) mixed repeated-measures ANOVA. Cognitive style was a between-subjects factor, whereas interpolated task and probe type were within-subjects factors. Significant interactions were followed up with simple-effects analyses. To further characterize VWM performance, hit rate, false-alarm rate, and sensitivity (d′) were additionally analyzed using the same three-way mixed ANOVA. Because d′ yielded the same pattern as accuracy, and false-alarm rate did not provide additional information beyond the main findings, the d′ and false-alarm rate results are reported in Appendix C. Furthermore, we calculated the difference between the task-absent and task-present conditions as the cost of consuming object-based attention by the interpolated task (Shen et al., 2015). Costs were analyzed using planned contrasts with paired-samples or independent-samples *t* tests to follow up interactions.

### 3.2 Results

The accuracy of the interpolated task across conditions in Experiments 2 and 3 was above 80%. There was no significant effect of either probe type or cognitive style on accuracy or reaction time in the interpolated task (see Appendix B for details).

#### 3.2.1 Accuracy

The main effect of the cognitive style was not significant [*M*_OVs_ = 0.79, 95% CI (0.76, 0.81) vs. *M*_SVs_ = 0.78, 95% CI (0.75, 0.80)], *F* (1, 38) = 0.38, *p* = 0.541, η_p_^2^ = 0.01. The main effect of the interpolated task was significant, *F* (1, 38) = 132.29, *p* < 0.001, η_p_^2^ = 0.78. Participants performed better in the task-absent [*M* = 0.84, 95% CI (0.82, 0.86)] than the task-present condition [*M* = 0.73, 95% CI (0.70, 0.75)]. The main effect of probe type was also significant, *F* (1, 38) = 240.85, *p* < 0.001, η_p_^2^ = 0.86, showing that the performance in the feature probe condition [*M* = 0.86, 95% CI (0.84, 0.88)] was better than that in the binding probe condition [*M* = 0.71, 95% CI (0.69, 0.72)].

The interaction between the cognitive style and the interpolated task was significant, *F* (1, 38) = 8.77, *p* = 0.005, η_p_^2^ = 0.19. A simple-effects analysis showed that OVs [*M* = 0.86, 95% CI (0.83, 0.88)] performed better than SVs [*M* = 0.82, 95% CI (0.79, 0.84)] in the task-absent condition, *F* (1, 38) = 5.55, *p* = 0.024, η_p_^2^ = 0.13, whereas they performed comparably in the task-present condition [*M*_OVs_ = 0.72, 95% CI (0.68, 0.75) vs. *M*_SVs_ = 0.74, 95% CI (0.70, 0.77)], *F* (1, 38) = 0.55, *p* = 0.462, η_p_^2^ = 0.01. The interaction between the interpolated task and the probe was also significant, *F* (1, 38) = 9.26, *p* = 0.004, η_p_^2^ = 0.20. A simple-effects analysis showed that in the feature probe condition, the accuracy in the task-absent condition [*M* = 0.90, 95% CI (0.88, 0.92)] was better than that in the task-present condition [*M* = 0.82, 95% CI (0.79, 0.85)], *F* (1, 38) = 52.36, *p* < 0.001, η_p_^2^ = 0.58. In the binding probe condition, the accuracy was also better in the task-absent condition [*M* = 0.77, 95% CI (0.75, 0.80)] than in the task-present condition [*M* = 0.64, 95% CI (0.61, 0.66)], *F* (1, 38) = 96.96, *p* < 0.001, η_p_^2^ = 0.72. Furthermore, the costs in the different probe conditions were analyzed by a planned contrast using a paired-samples *t*-test. The result showed that the cost in the binding probe condition [*M* = 0.14, 95% CI (0.11, 0.17)] was larger than that in the feature probe condition [*M* = 0.09, 95% CI (0.06, 0.11)], *t* (39) = 2.80, *p* = 0.008, Cohen’s *d* = 0.90. The interaction between the cognitive style and the probe was not significant, *F* (1, 38) = 2.62, *p* = 0.114, η_p_^2^ = 0.06.

The three-way interaction was significant, *F*(1, 38) = 7.97, *p* = 0.008, η_p_^2^ = 0.17. Simple-simple-effects analyses showed that in the feature task-absent condition, the difference between OVs and SVs was not significant, *F*(1, 38) = 0.00, *p* = 0.980, η_p_^2^ = 0.00. In the feature task-present condition, the difference between OVs and SVs was not significant, *F*(1, 38) = 0.11, *p* = 0.741, η_p_^2^ = 0.00. In the binding task-absent condition, OVs [*M* = 0.81, 95% CI (0.78, 0.84)] showed higher accuracy than SVs, *F*(1, 38) = 14.08, *p* < 0.001, η_p_^2^ = 0.27. In the binding task-present condition, the difference between OVs and SVs was not significant, *F*(1, 38) = 0.96, *p* = 0.335, η_p_^2^ = 0.03 (see Figure 6b).

**Figure 6:**
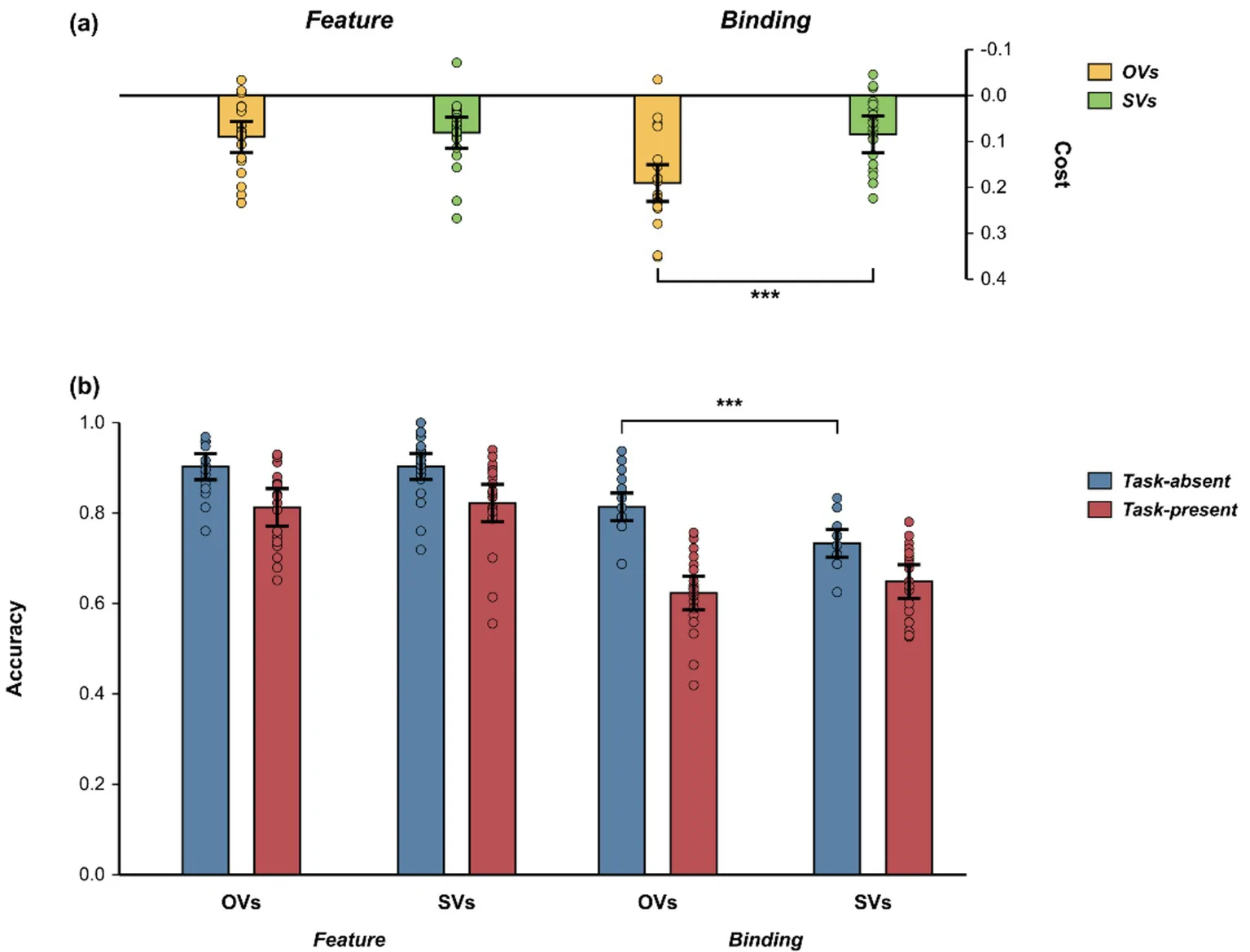
Performance accuracy in Experiment 2. Each dot corresponds to the data of an individual participant. **(a)** The cost of the interpolated task. The cost was calculated as the difference in accuracy between the task-absent and task-present conditions (task-absent minus task-present). **(b)** Response accuracy in each condition for OVs and SVs. Bars represent the 95% CIs, *** *p <* 0.001.

To follow up the significant three-way interaction, task costs were compared between OVs and SVs separately for feature and binding probes using independent-samples *t* tests. For feature probes, the cost did not differ significantly between OVs and SVs, *t* (38) = 0.38, *p* = 0.703, Cohen’s d = 0.12. For binding probes, the cost was greater for OVs than for SVs, *t* (38) = 3.79, *p* < 0.001, Cohen’s d = 1.20 (see Figure 6a).

#### 3.2.2 Hit rate

The main effect of cognitive style was significant, *F* (1, 38) = 9.59, *p* = 0.004, η_p_^2^ = 0.20. The hit rate was higher for OVs [*M* = 0.82, 95% CI (0.78, 0.86)] than for SVs [*M* = 0.74, 95% CI (0.70, 0.78)]. The main effect of interpolated task was significant, *F* (1, 38) = 56.51, *p* < 0.001, η_p_^2^ = 0.60. The hit rate was higher in the task-absent condition [*M* = 0.84, 95% CI (0.82, 0.86)] than in the task-present condition [*M* = 0.72, 95% CI (0.68, 0.76)]. The main effect of probe type was significant, *F* (1, 38) = 75.14, *p* < 0.001, η_p_^2^ = 0.66. The hit rate was higher in the feature condition [*M* = 0.86, 95% CI (0.84, 0.88)] than in the binding condition [*M* = 0.70, 95% CI (0.66, 0.74)].

The interaction between cognitive style and interpolated task was not significant, *F* (1, 38) = 1.10, *p* = 0.301, η_p_^2^ = 0.03. The interaction between cognitive style and probe type was significant, *F* (1, 38) = 10.79, *p* = 0.002, η_p_^2^ = 0.22. Simple-effects analyses showed the following pattern. In the feature condition, the difference between OVs [*M* = 0.87, 95% CI (0.84, 0.90)] and SVs [*M* = 0.85, 95% CI (0.82, 0.88)] was not significant, *F* (1, 38) = 0.70, *p* = 0.407, η_p_^2^ = 0.02. In the binding condition, OVs (*M* = 0.77, 95% CI [0.72, 0.83]) showed a higher hit rate than SVs (*M* = 0.63, 95% CI [0.58, 0.69]), *F* (1, 38) = 13.64, *p* < 0.001, η_p_^2^ = 0.26. The interaction between interpolated task and probe type was not significant, *F* (1, 38) = 0.91, *p* = 0.347, η_p_^2^ = 0.02.

The three-way interaction was significant, *F* (1, 38) = 4.95, *p* = 0.032, η_p_^2^ = 0.12. Simple-simple-effects analyses showed that in the feature task-absent condition, the difference between OVs [*M* = 0.92, 95% CI (0.89, 0.94)] and SVs [*M* = 0.91, 95% CI (0.89, 0.94)] was not significant, *F* (1, 38) = 0.05, *p* = 0.817, η_p_^2^ = 0.00. In the feature task-present condition, the difference between OVs [*M* = 0.82, 95% CI (0.77, 0.88)] and SVs [*M* = 0.79, 95% CI (0.73, 0.84)] was not significant, *F* (1, 38) = 0.82, *p* = 0.370, η_p_^2^ = 0.02. In the binding task-absent condition, OVs (*M* = 0.86, 95% CI [0.82, 0.91]) showed a higher hit rate than SVs [*M* = 0.68, 95% CI (0.63, 0.72)], *F* (1, 38) = 34.66, *p* < 0.001, η_p_^2^ = 0.48. In the binding task-present condition, the difference between OVs [*M* = 0.68, 95% CI (0.60, 0.76)] and SVs [*M* = 0.59, 95% CI (0.51, 0.67)] was not significant, *F* (1, 38) = 2.64, *p* = 0.112, η_p_^2^ = 0.07 (see Figure 7b).

**Figure 7:**
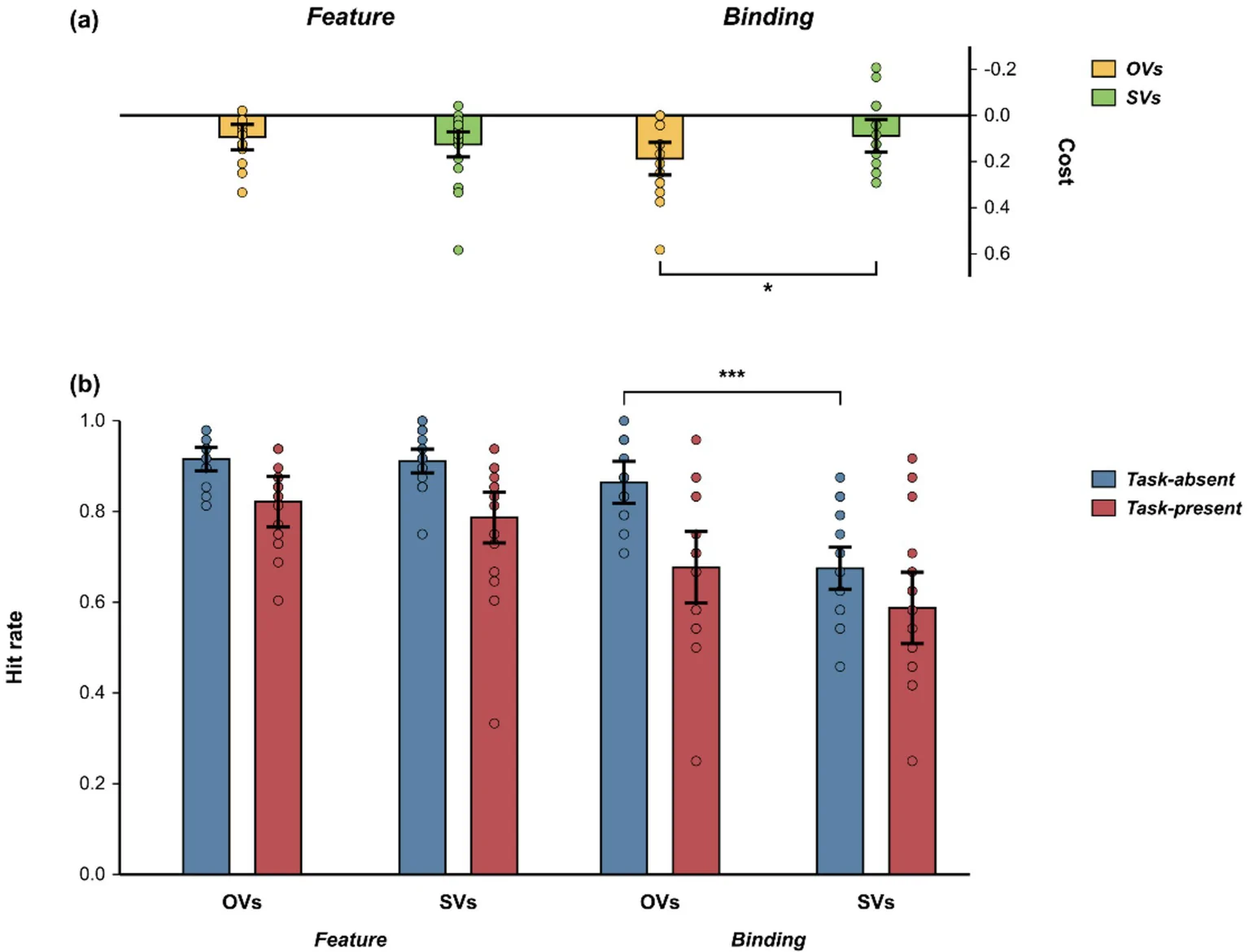
Hit rate in Experiment 2. Each dot corresponds to the data of an individual participant. **(a)** The cost of the interpolated task. The cost was calculated as the difference in hit rate between the task-absent and task-present conditions (task-absent minus task-present). **(b)** Hit rate in each condition for OVs and SVs. Bars represent the 95% CIs, * *p <* 0.05, *** *p <* 0.001.

To follow up the significant three-way interaction, task costs were compared between OVs and SVs separately for feature and binding probes using independent-samples *t* tests. For feature probes, the cost did not differ significantly between OVs and SVs [OVs: *M* = 0.09, 95% CI (0.04, 0.15); SVs: *M* = 0.13, 95% CI (0.07, 0.18)], *t* (38) = −0.81, *p* = 0.422, Cohen’s *d* = −0.26. For binding probes, the cost was greater for OVs than for SVs [OVs: *M* = 0.19, 95% CI (0.12, 0.26); SVs: *M* = 0.09, 95% CI (0.02, 0.16)], *t* (38) = 2.03, *p* = 0.049, Cohen’s *d* = 0.64 (see Figure 7a).

### 3.3 Discussion

In Experiment 2, we investigated whether OVs and SVs differ in their dependence on object-based attention in holding bindings in VWM. During the maintenance of features and bindings in VWM, participants answered two questions about the box-line stimuli in the interpolated task, which consumed object-based attention.

For binding probes, OVs were more accurate than SVs in the task-absent condition, which replicated the findings of Experiment 1, while this advantage of OVs disappeared when an interpolated task was introduced. In contrast, for feature probes (color/shape), the VWM accuracy of SVs and OVs was comparable regardless of whether the interpolated task was presented or not. Furthermore, we analyzed the cost of consuming object-based attention by considering the difference between the VWM performance with and without the interpolated task. The results showed that OVs had a larger cost than SVs when holding bindings, while OVs and SVs had comparable costs when holding features. The same pattern was observed for d′ and hit rate. These findings extend previous work (Gao and Shen, 2024; Gao et al., 2017; Shen et al., 2015; Zhou et al., 2021) by showing that the selective interference of object-based attention-demanding tasks with VWM binding maintenance varies with visualizer cognitive style. How do OVs and SVs allocate attentional resources differently before and after the interpolated task stages in processing bindings in VWM? In Experiment 3, we explored this issue using ERP technology.

## 4 Experiment 3: how do OVs and SVs invest resources in processing bindings across distinct VWM stages? Evidence from ERPs

### 4.1 Method

#### 4.1.1 Participants

An *a priori* power analysis was conducted using the same parameters as in Experiment 2, except for two repeated measurements. The analysis was based on the behavioral effect from the related paradigm, as comparable ERP-specific effect-size estimates were unavailable. The estimated minimum sample size was 16 per group. Forty students were randomly selected from the remaining OV and SV pools. Five participants were excluded due to either an insufficient number of artifact-free trials in the electroencephalography (EEG) recordings (*n* = 3) or malfunctioning of the EEG recorder (*n* = 2), leaving 18 OVs [13 females, 18–21 years old; *M*_object-scores_ = 3.81, 95% CI (3.69, 3.93); *M*_spatial-score_ = 2.33, 95% CI (2.18, 2.47)] and 17 SVs [8 females, 18–22 years old; *M*_object-scores_ = 2.67, 95% CI (2.48, 2.86); *M*_spatial-score_ = 3.40, 95% CI (3.25, 3.55)], for whom the data were analyzed. The two groups did not differ in gender distribution (*χ*^2^ = 2.31, *p* = 0.129).

#### 4.1.2 Materials and apparatus

The stimuli, apparatus and software were the same as those used in Experiment 2.

#### 4.1.3 Design and procedure

A 2 (cognitive style: *OVs* vs. *SVs*) × 2 (interpolated task: *task-present* vs. *task-absent*) mixed design was utilized, in which cognitive style served as a between-subjects factor and the interpolated task served as a within-subjects factor. There were two blocks: the binding probe with the interpolated task absent or present.

Participants were seated in a quiet room approximately 57 cm from the screen. The procedure was modified from the binding probe condition of Experiment 2 (see Figure 8); the modifications were as follows. First, the duration of fixation varied between 400 ms and 600 ms to avoid an anticipation effect. Second, the durations of the feature report question and the probe were fixed at 2000 ms to equate the duration of each trial for the EEG analysis. Third, to avoid excessively long intervals in VWM, the number of feature report questions in the interpolated task was reduced to one, which was randomly selected from the two options (i.e., either the box or the line). Otherwise, the experimental setting was identical to that in the binding probe condition of Experiment 1. Each block contained 50 trials and was presented twice, resulting in a total of 200 trials. Participants completed 20 practice trials before each block. The order of the two blocks was counterbalanced between participants. A 10 min break was given between the blocks. The task lasted approximately 60 min.

**Figure 8:**
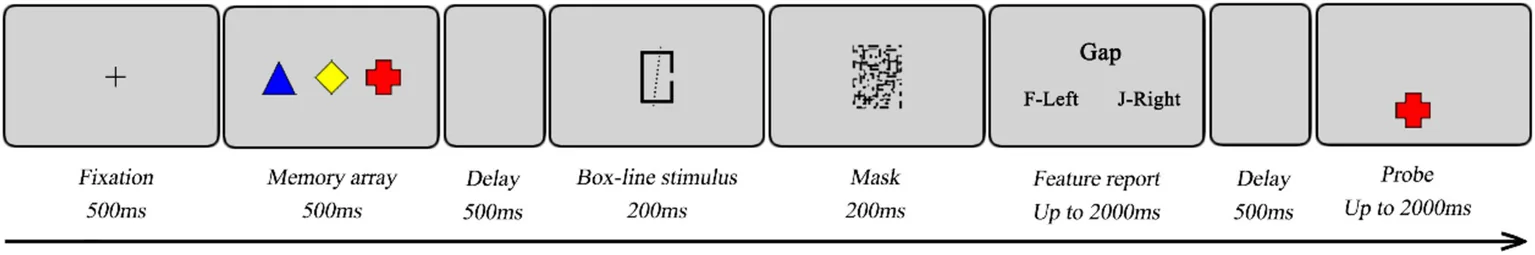
A schematic illustration of a trial in Experiment 3 (in this trial the probe was present in the memory array).

#### 4.1.4 Behavioral analysis

Accuracy and hit rate were analyzed separately using 2 (cognitive style) × 2 (interpolated task) two-way mixed repeated-measures ANOVAs. The analyses were the same as those used in Experiment 2.

#### 4.1.5 Electroencephalogram recording and analysis

##### 4.1.5.1 Recording

EEG signals were recorded using 64-channel Ag/AgCl electrodes that were mounted in an elastic cap (NeuroScan, El Paso, Texas, USA) according to the 10–20 system, with the reference on the left mastoid and a ground electrode on the medial frontal aspect. A vertical electrooculogram (VEOG) was recorded from electrodes located 1 cm below and above the left eye. The horizontal EOG (HEOG) was recorded as the left versus the right orbital rim. VEOG was used to detect blink artifacts, and HEOG was used to detect horizontal eye movement artifacts. The impedances of all electrodes were maintained at less than 5 kΩ. The signals were digitized at 1000 Hz.

##### 4.1.5.2 Preprocessing

Offline analysis was conducted using the EEGLAB toolbox in MATLAB (The MathWorks, Inc., Natick, MA, USA). The data were first down-sampled to 500 Hz to save storage space and filtered with an IIR Butterworth filter with a 0.5–30 Hz bandpass. The data were subsequently re-referenced to the 1/2 voltage value of the right mastoid and were epoched from −200 ms to 3,000 ms around the onset of the memory array. Epochs with incorrect behavioral responses on either the interpolated task or the VWM task or with non-stereotyped artifacts, such as those induced by generalized head or electrode movements, were excluded. Epochs were baseline-normalized using the whole epoch as the baseline to improve independent component analysis (ICA; Groppe et al., 2009). Through visual inspection of the ICA results, we identified components that captured eye blinks and horizontal eye movements. These components were removed from the EEG data; on average, 2.43 ± 0.24 ICs were removed. The post-ICA epochs were baseline-corrected and underwent automatic artifact rejection based on a threshold of ±100 μV for the time range of −200–3,000 ms. Following data processing, participants with fewer than 40 trials remaining per condition were excluded from the analysis. For the included participants, an average of 75.4 trials in the task-absent condition and 56.2 trials in the task-present condition were retained per participant, corresponding to an overall trial rejection rate of 34.2% due to incorrect behavioral responses and artifact detection. The rejection rate did not differ significantly between OVs (32.6%) and SVs (35.8%), *t* (32.88) = − 0.96, *p* = 0.342, Cohen’s *d* = − 0.33.

##### 4.1.5.3 ERP analysis

The epochs were baseline-corrected using a period from −200 ms to 0 ms relative to the onset of the memory array and averaged for each condition to obtain grand average waveforms. Based on previous ERP studies (Gunseli et al., 2014b; Pinal et al., 2015; Ruchkin et al., 1990; Wolach and Pratt, 2001), the scalp regions and broad latency ranges of the P2 and LPC were defined *a priori*. The measurement windows were refined based on the grand-average waveforms collapsed across all participants and experimental conditions (Keil et al., 2014). In the present study, the mean amplitude of P2 was calculated at the electrode sites of Fz and FCz at 160–200 ms (Pinal et al., 2015; Wolach and Pratt, 2001). The mean amplitude of LPC was calculated at the electrode sites of Pz and POz at 2000–2250 ms during the interpolated task period, that is, 600–850 ms after the presentation of the feature report question (Gunseli et al., 2014a,b). The mean amplitudes of P2 and LPC were analyzed separately using a two-way mixed repeated-measures ANOVA.

### 4.2 Results

#### 4.2.1 Accuracy

The main effect of cognitive style was not significant, *F* (1, 33) = 3.55, *p* = 0.068, η_p_^2^ = 0.10. Accuracy did not differ significantly between OVs [*M* = 0.75, 95% CI (0.72, 0.78)] and SVs [*M* = 0.71, 95% CI (0.68, 0.74)]. The main effect of interpolated task was significant, *F* (1, 33) = 139.12, *p* < 0.001, η_p_^2^ = 0.81. Accuracy was higher in the task-absent condition [*M* = 0.78, 95% CI (0.76, 0.81)] than in the task-present condition [*M* = 0.67, 95% CI (0.65, 0.70)]. The interaction between cognitive style and interpolated task was not significant, *F* (1, 33) = 3.21, *p* = 0.082, η_p_^2^ = 0.09.

#### 4.2.2 Hit rate

The main effect of cognitive style was not significant, *F* (1, 33) = 0.46, *p* = 0.504, η_p_^2^ = 0.01. The hit rate did not differ significantly between OVs [*M* = 0.70, 95% CI (0.64, 0.77)] and SVs [*M* = 0.73, 95% CI (0.67, 0.80)]. The main effect of interpolated task was significant, *F* (1, 33) = 64.52, *p* < 0.001, η_p_^2^ = 0.66. The hit rate was higher in the task-absent condition [*M* = 0.78, 95% CI (0.73, 0.82)] than in the task-present condition (*M* = 0.66, 95% CI [0.61, 0.71]).

The interaction between cognitive style and interpolated task was significant, *F* (1, 33) = 9.04, *p* = 0.005, η_p_^2^ = 0.22. Simple-effects analyses showed that in the binding task-absent condition, the difference between OVs [*M* = 0.78, 95% CI (0.72, 0.84)] and SVs [*M* = 0.77, 95% CI (0.71, 0.83)] was not significant, *F* (1, 33) = 0.10, *p* = 0.749, η_p_^2^ = 0.00. In the binding task-present condition, the difference between OVs [*M* = 0.63, 95% CI (0.56, 0.69)] and SVs [*M* = 0.70, 95% CI (0.63, 0.77)] was not significant, *F* (1, 33) = 2.23, *p* = 0.145, η_p_^2^ = 0.06. To follow up the significant interaction, task cost was calculated and compared between OVs and SVs using an independent-samples planned *t* test. The cost was greater for OVs [*M* = 0.16, 95% CI (0.12, 0.20)] than for SVs [*M* = 0.07, 95% CI (0.03, 0.11)], *t* (33) = 3.01, *p* = 0.005, Cohen’s *d* = 1.02.

#### 4.2.3 ERP results

##### 4.2.3.1 P2

A 2 cognitive style × 2 interpolated task two-way mixed, repeated-measures ANOVA on the P2 was conducted. The main effect of the interpolated task was not significant [Task-absent: *M* = 4.30 μV, 95% CI (3.08, 5.52); Task-present: *M* = 3.91 μV, 95% CI (2.67, 5.15)], *F* (1, 33) = 1.79, *p* = 0.190, η_p_^2^ = 0.05. The main effect of cognitive style was not significant either [OVs: *M* = 4.89 μV, 95% CI (3.22, 6.56); SVs: *M* = 3.32 μV, 95% CI (1.60, 5.03)], *F* (1, 33) = 1.80, *p* = 0.189, η_p_^2^ = 0.05. However, the interaction was significant, *F* (1, 33) = 4.60, *p* = 0.039, η_p_^2^ = 0.12. A simple-effects analysis showed that for OVs the P2 was smaller in the task-present condition than that in the task-absent condition, *F* (1, 33) = 6.24, *p* = 0.018, η_p_^2^ = 0.16. On the other hand, for SVs no difference was observed between the task-absent and -present conditions, *F* (1, 33) = 0.32, *p* = 0.578, η_p_^2^ = 0.01 (see Figures 9, 10).

**Figure 9:**
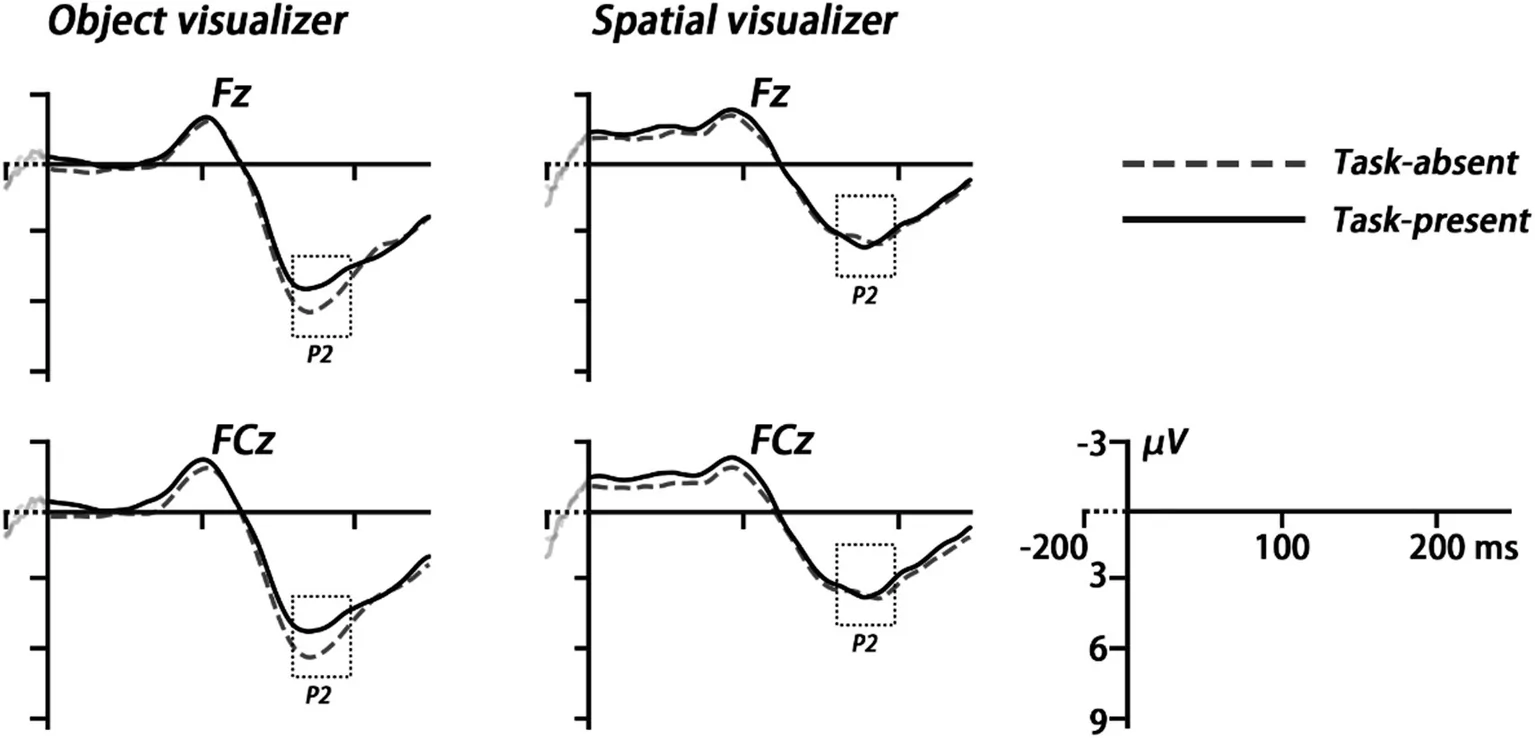
The grand-averaged waveforms during VWM encoding. Averaged P2 waveforms for OVs and SVs in the task-absent and task-present conditions are shown at the Fz and FCz electrode sites. The rectangles show the time windows (from 160 ms to 200 ms after the onset of the memory array) used for the statistical analyses.

**Figure 10:**
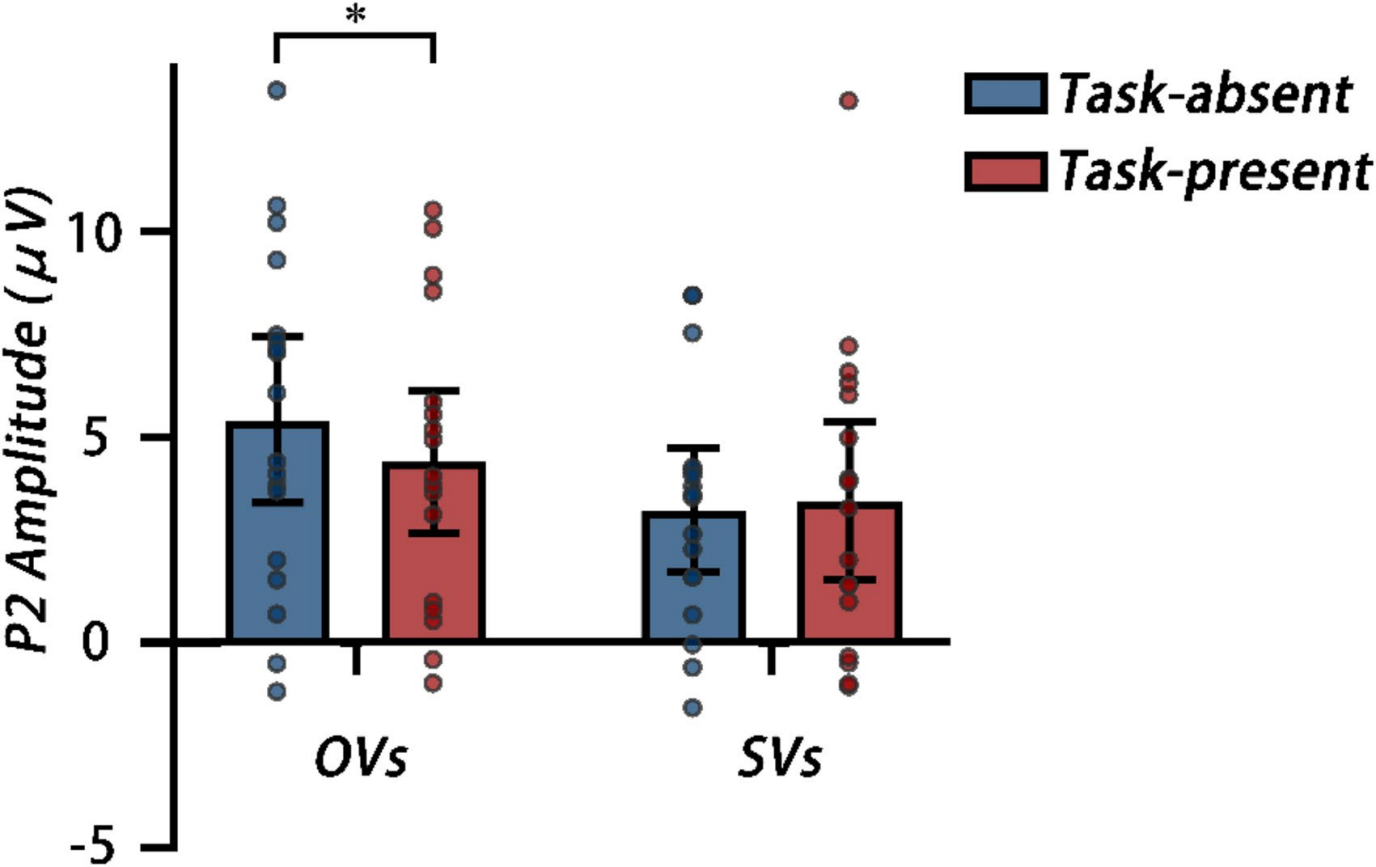
Mean P2 amplitudes from 160 ms to 200 ms after the memory array onset across the Fz and FCz electrode sites for the task-absent and task-present conditions; each dot corresponds to the data of an individual participant. Bars represent the 95% CIs, *p < 0.05.

##### 4.2.3.2 LPC

A 2 cognitive style × 2 interpolated task two-way mixed, repeated-measures ANOVA on the LPC was conducted. The main effect of the interpolated task was significant, *F* (1, 33) = 18.58, *p* < 0.001, η_p_^2^ = 0.36, showing that the LPC in the task-present condition [*M* = 2.18 μV, 95% CI (1.32, 3.04)] was larger than that in the task-absent condition [*M* = 0.76 μV, 95% CI (0.18, 1.34)]. The main effect of cognitive style was not significant [OVs: *M* = 1.82 μV, 95% CI (0.91, 2.74); SVs: *M* = 1.12 μV, 95% CI (0.18, 2.06)], *F* (1, 33) = 1.20, *p* = 0.281, η_p_^2^ = 0.04. However, the interaction was significant, *F* (1, 33) = 4.66, *p =* 0.038, η_p_^2^ = 0.12. A simple-effects analysis showed that for OVs the LPC was larger in the task-present condition than in the task-absent condition, *F* (1, 33) = 21.54, *p* < 0.001, η_p_^2^ = 0.40. On the other hand, for SVs no difference was observed between the two conditions, *F* (1, 33) = 2.25, *p* = 0.143, η_p_^2^ = 0.06 (see Figures 11, 12).

**Figure 11:**
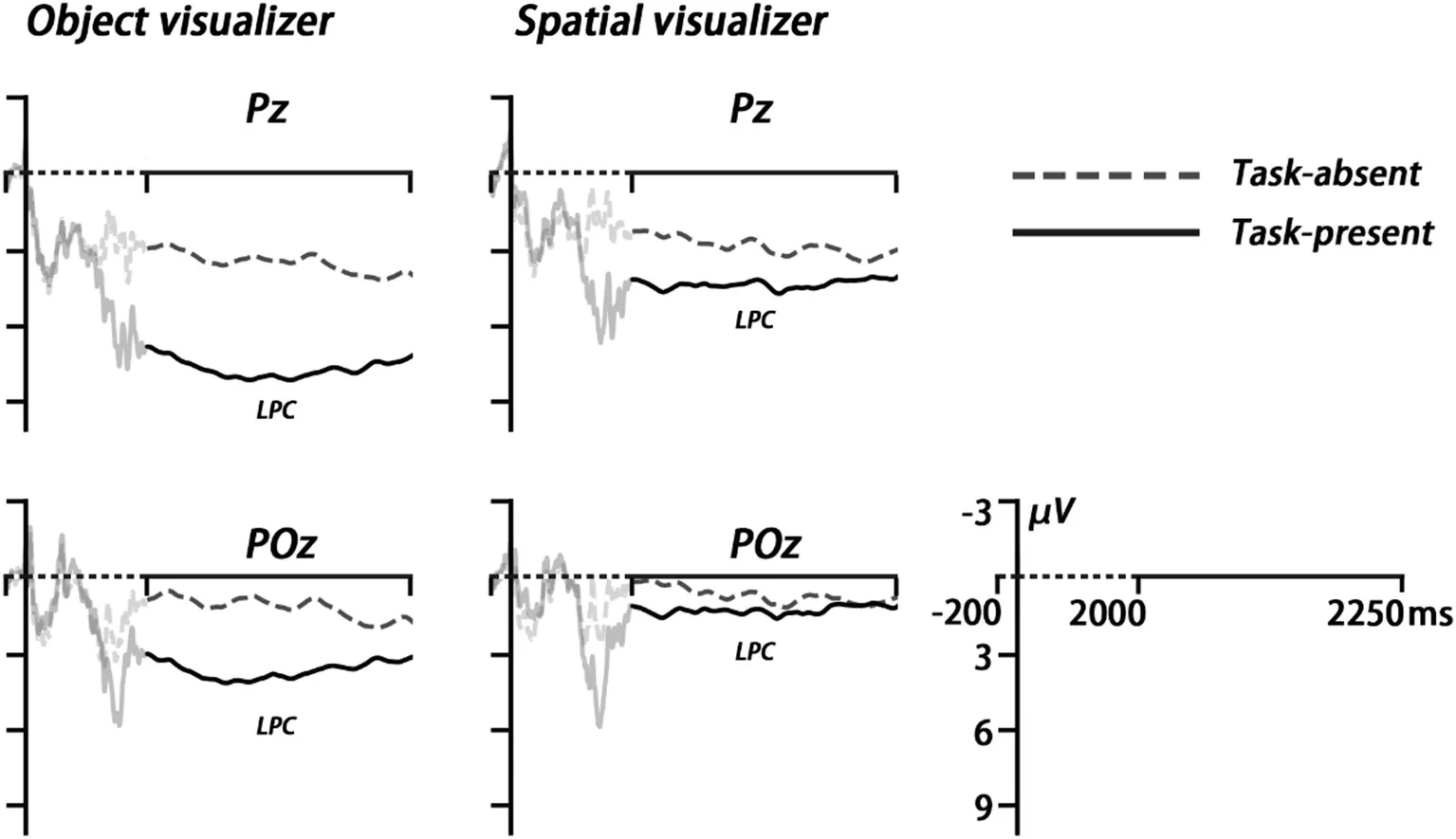
The grand-averaged waveforms during the interpolated task performance. Averaged LPC waveforms for OVs and SVs in the task-absent and task-present conditions are shown at the Pz and POz electrode sites. The time window from 2000 ms to 2,250 ms after the onset of the memory array were used for the statistical analyses. The LPC time window was displayed on an expanded horizontal scale relative to the preceding interval (−200 to 2000 ms).

**Figure 12:**
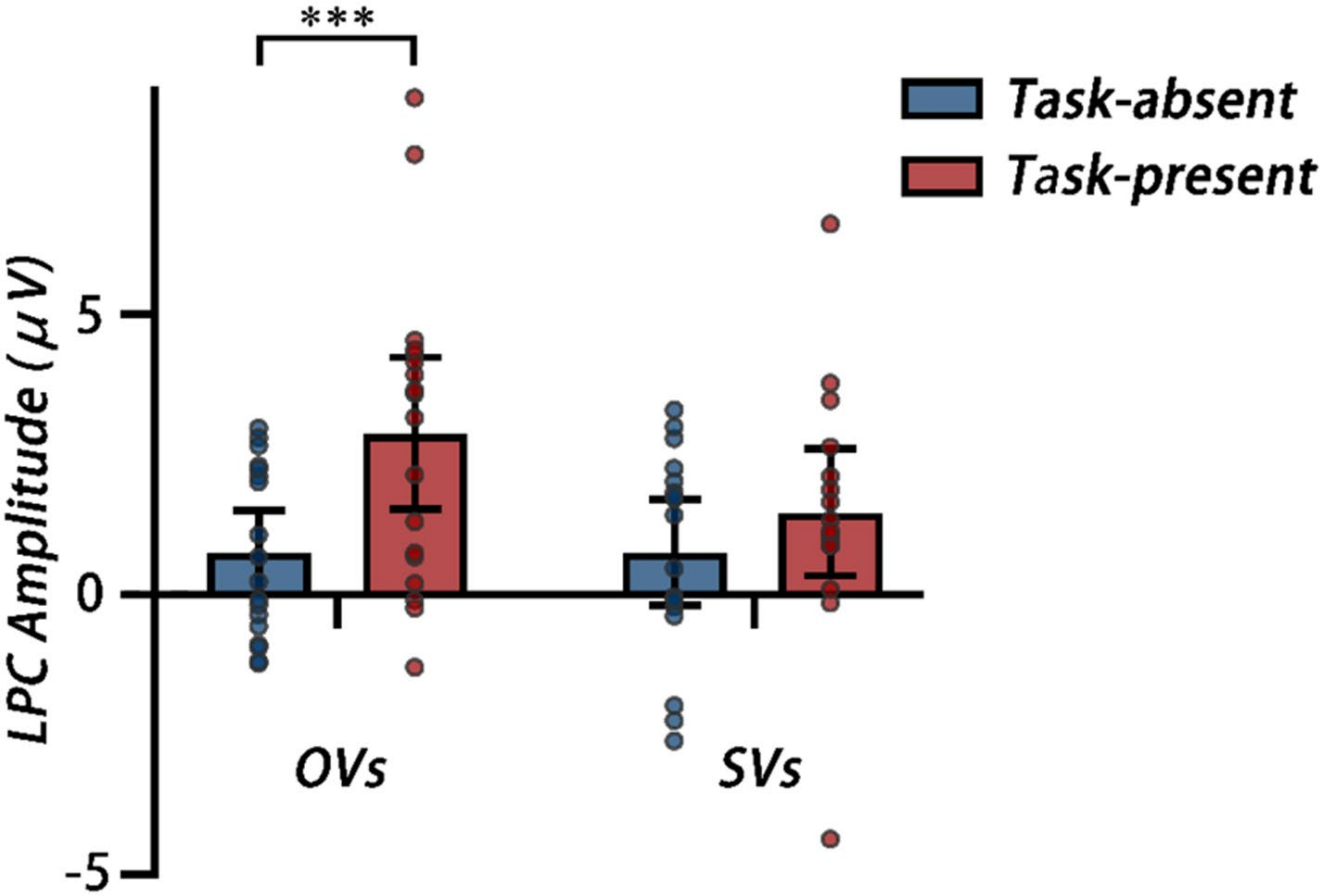
Mean LPC amplitudes from 2000 ms to 2,250 ms after the memory array onset across the Pz and POz electrode sites for the task-absent and task-present conditions; each dot corresponds to the data of an individual participant. Bars represent the 95% CIs, ***p < 0.001.

### 4.3 Discussion

In Experiment 3, we investigated neural correlates of attentional resource allocation for OVs and SVs when they processed bindings in VWM. As the interpolated task, participants answered one question about the box or the line during the maintenance of bindings in VWM. ERPs were recorded during the VWM encoding and the interpolated task performance. The behavioral results showed that only the hit-rate finding was consistent with that in Experiment 2, with OVs showing a larger cost of the interpolated task than SVs. The ERP results showed that when holding bindings in VWM, OVs evoked a smaller P2 during VWM encoding and a larger LPC during the interpolated task performance in the task-present condition compared to the task-absent condition, while SVs evoked comparable P2 and LPC amplitudes under both conditions. These divergent neural patterns between OVs and SVs suggest that OVs and SVs differ in how they engage resources when processing bindings before and after the interpolated task stages in VWM.

## 5 General discussion

In the present study, we explored whether the visualizer cognitive style modulates the operation of object-based attention in processing bindings in VWM. The results of the behavioral experiments showed that, compared to SVs, OVs maintained bindings more stably and incurred a larger cost of holding bindings when the interpolated task consumed object-based attention. The results of the ERP experiment showed that, compared to SVs, OVs elicited a smaller P2 during VWM encoding and a larger LPC during the interpolated task performance in the task-present condition than in the task-absent condition.

### 5.1 Visualizer cognitive style modulates the reliance on object-based attention in holding bindings in VWM

The two behavioral experiments revealed that OVs maintained bindings more stably. Moreover, with the introduction of the interpolated task, OVs incurred a larger cost than SVs in maintaining bindings in VWM, whereas OVs and SVs showed comparable costs for features. When performing the interpolated task, participants are required to select a target object from two spatially overlapping objects and report its features, such that successful performance places substantial demands on object-based attention (Chen, 2012; Duncan, 1984). According to feature integration theory (Bouvier and Treisman, 2010; Treisman, 1996), object-based attention supports the rehearsal of bindings during VWM maintenance through a re-entry process that verifies binding correctness based on bottom-up signals and consolidates a more stable binding representation. This process is required for bindings, but not for features. When object-based attention is occupied by the interpolated task, the re-entry process is disrupted, resulting in greater impairment of bindings than of features (Gao and Shen, 2024; Shen et al., 2015). In the present study, the larger cost for OVs suggests that disruption of object-based attention had a greater impact on the re-entry process that supports their binding maintenance. Therefore, we propose that OVs rely more on object-based attention than SVs during binding maintenance in VWM, extending previous findings (Gao and Shen, 2024; Shen et al., 2015) by suggesting that the role of object-based attention in binding maintenance may vary with visualizer cognitive style.

Why did OVs rely more on object-based attention than SVs during the maintenance of bindings in VWM? On the one hand, OVs and SVs have different visual processing styles. OVs tend to encode multiple parts of an object as a single unit and process information holistically, whereas SVs focus on discrete parts of an object and process information analytically (for a review, see Kozhevnikov and Blazhenkova, 2013). According to the integrated competition model (Duncan et al., 1997), when an individual directs object-based attention to a feature of a multiple-feature object, activity in the neural regions dealing with that feature will first be enhanced and then spread to neural regions dealing with the other features within that object. Accordingly, less object-based attention may induce lower neural activity in dealing with features of that object, leading to an unstable VWM representation. Compared to SVs, OVs who process information holistically could more easily integrate multiple features into one object, depending more on object-based attention, thereby forming more stable bindings.

On the other hand, OVs and SVs differ in the relative efficiency of the neural pathways responsible for processing bindings. It was found that OVs have greater efficiency in the ventral pathway in object-related tasks, compared to SVs (Motes et al., 2008). The ventral pathway is responsible for object processing and is relatively more involved in object-based attention through the re-entry process, promoting the stability of objects (Cavanagh et al., 2023; Cohen and Frank, 2015). Binding representations are fragile in VWM (Hitch et al., 2019); thus, involving object-based attention through the re-entry process consolidates their stability. In the present study, compared to SVs, OVs with greater efficiency in the re-entry process may rely more on object-based attention during the maintenance of bindings in VWM, resulting in more stable binding representations.

According to the activity-silent working memory framework, working memory representations can temporarily shift from an active to a passive state when they are not immediately task-relevant and can subsequently be reactivated when they become relevant again (Stokes, 2015; Zhang et al., 2022). In the present study, the interpolated task may have temporarily shifted the maintained VWM representations into a passive state, with the subsequent state transition or reactivation contributing to the observed memory cost. The activity-silent working memory framework may explain the general memory cost induced by the interpolated task. It would be interesting for future research to examine whether this framework can also account for differences between visualizer cognitive styles.

### 5.2 Visualizer cognitive style modulates the allocation of object-based attention for binding processes at distinct stages in VWM

In Experiment 3, participants were required to maintain bindings in VWM with or without an interpolated task. P2 was recorded during the presentation of the memory items. The results showed that OVs elicited a smaller P2 during VWM encoding in the task-present condition than in the task-absent condition, whereas SVs elicited comparable P2 amplitudes across conditions.

P2 serves as a neural marker of attentional allocation in VWM (Pinal et al., 2015; Smith, 1993; Wolach and Pratt, 2001). In Smith’s (1993) study, participants were required to remember words in the ‘study-phase’ and then judge whether or not those words had been previously studied in the ‘test-phase’. During the ‘study-phase’, the P2 was larger for words that were correctly recognized during the ‘test-phase’ compared to those that were not. The researcher proposed that P2 might be involved in the engagement of attentional or working memory resources. In another study (Wolach and Pratt, 2001), participants were sequentially presented with two memory digits and two distractors. P2 was recorded during the presentation of both memory digits and distractors. The memory digits elicited a larger P2 than the distractors. The larger P2 indicated increased allocation of attention to the memory digits compared to the distractors. Furthermore, Pinal et al. (2015) found that P2 reflects an early top-down mechanism for the rapid evaluation of stimulus significance, and a reduction in its amplitude indicates a decrease in the allocation of processing resources during the encoding of information in VWM. These findings indicate that the frontocentral P2 reflects the allocation of attention during the encoding stage of VWM. Therefore, our findings reveal that when less attention is allocated during the encoding of bindings in VWM in the task-present condition than in the task-absent condition, OVs rely on object-based attention and thus show a smaller P2, whereas SVs, who do not show the same reliance on object-based attention, do not exhibit comparable task-related modulation.

Earlier work linked LPC to VWM retention and the processing demand associated with VWM performance (García-Larrea and Cézanne-Bert, 1998; Ruchkin et al., 1990). Subsequent studies have pointed to a relationship between LPC and the effort invested in performing working memory tasks (Gunseli et al., 2014a,b). In one study (Gunseli et al., 2014b), participants were required to maintain VWM representations for a subsequent simple search task (target color was different from that of distractors) and a difficult search task (target color was the same as that of distractors). A larger LPC was evoked in the difficult than in the simple search task, indicating that LPC indexes investment of effort during VWM maintenance. In dual-task studies, LPC has further been shown to be sensitive to the allocation of attentional and cognitive resources during secondary task processing while information is concurrently maintained in working memory (Jongen and Jonkman, 2011; Pratt et al., 2011). For example, Jongen and Jonkman (2011) required participants to perform a Stroop task during the maintenance interval of a working memory task and found a larger LPC for incongruent than congruent trials. This suggests that LPC reflects greater engagement of cognitive resources during the more demanding secondary task condition. In the present study, participants were required to complete an interpolated task while retaining bindings in VWM. The interpolated task increased the overall task difficulty while competing for limited resources with the binding maintenance process. In these circumstances, OVs showed greater engagement of processing resources in response to the demands of both maintaining the bindings and performing the interpolated task, manifesting as a larger LPC. On the other hand, SVs showed relatively consistent processing effort regardless of the presence or absence of the interpolated task, manifesting as similar LPC amplitudes across the task conditions.

According to the model of the cross-level iterative re-entry, visual processing involves iterative communication between different levels of brain regions (Lamme and Roelfsema, 2000; Spalek et al., 2024), a process supporting the deployment of object-based attention to form bindings during visual perception (Cavanagh et al., 2023; Koivisto and Silvanto, 2011). In VWM binding maintenance, object-based attention underlies the rehearsal of bindings in VWM through a re-entry process (Gao and Shen, 2024; Shen et al., 2015). Consistent with this framework, OVs showed a larger decline in binding performance when the interpolated task placed demands on object-based attention and also exhibited distinct ERP modulations across different stages of VWM processing, suggesting that the involvement of object-based attention in supporting VWM binding may vary flexibly across processing stages.

In general, compared to SVs, OVs maintain bindings more stably, rely more on object-based attentional resources, and differ from SVs in the engagement of neural resource across different stages of VWM processing. Together, these findings suggest that the contribution of object-based attention to VWM binding maintenance depends on how individuals tend to represent visual information and extends across different stages of VWM processing.

### 5.3 Limitations

Several limitations should be noted. First, the present study adopted an extreme-group design to compare individual differences. Future studies should examine visualizer cognitive style continuously in unselected samples. Second, the task-present condition in Experiment 2 resulted in a longer VWM retention interval than the task-absent condition. Although additional analyses suggested that this difference in retention duration did not account for the critical effects observed in the present study (see Appendix D), future studies should more closely equate retention duration across conditions. Third, baseline performance was lower for bindings than for features, which may reflect differences in task difficulty and discriminability between the two conditions. Although this baseline discrepancy may not fully account for the individual differences of primary interest in the present study, future studies should take it into account. Finally, articulatory suppression was not used in Experiments 2 and 3 to avoid introducing an additional concurrent task and increasing general processing load. Thus, verbal encoding cannot be completely ruled out and should be examined more directly in future studies.

## Data Availability

The datasets presented in this study can be found in online repositories. The names of the repository/repositories and accession number(s) can be found at: https://osf.io/f69rh/?view_only=a7d33b50165848bea8a562fe8f66e554.
