# Supplementary material for: Visualizer cognitive style modulates the operation of object-based attention in processing bindings in visual working memory
Source: Front Psychol. 2026 Oct 12;17:1875838. doi: 10.3389/fpsyg.2026.1875838 (PMC13696248; doi:10.3389/fpsyg.2026.1875838)
Supplement: Supplementary file 1 [file Supplementary_file_1.pdf]

Supplementary Appendix

Appendix A Separate Analyses for Color, Shape, and Binding Conditions

Accuracy data in each condition were analyzed using a 2 (cognitive style) × 2 (interpolated task) × 3 (probe: *color*, *shape*, *binding*) three-way mixed, repeated-measures ANOVA, with post hoc pairwise comparisons corrected using the Bonferroni method.

The main effect of the interpolated task was significant, F (1, 38) = 114.57, $p < 0.001$, $\eta_p^2 = 0.75$. Participants performed better in the task-absent (M = 0.86, 95% CI [0.84, 0.88]) than the task-present condition (M = 0.76, 95% CI [0.73, 0.78]). The main effect of probe was also significant, F (2, 76) = 95.70, $p < 0.001$, $\eta_p^2 = 0.72$, showing that the performance in the color probe condition (M = 0.89, 95% CI [0.86, 0.91]) was better than that in the shape probe condition (M = 0.84, 95% CI [0.81, 0.86]), p = 0.006, and that in the binding probe condition (M = 0.71, 95% CI [0.69, 0.72]), $p < 0.001$; the performance in the shape probe condition was also better than that in the binding condition ($p < 0.001$). The main effect of the cognitive style was not significant, F (1, 38) = 0.09, p = 0.765, $\eta_p^2 = 0.00$.

The interaction between the cognitive style and the interpolated task was significant, F (1, 38) = 4.61, p = 0.038, $\eta_p^2 = 0.11$. A simple-effects analysis showed that both OVs and SVs performed better in the task-absent condition (M_{OVs} = 0.87, 95% CI [0.85, 0.90], M_{SVs} = 0.85, 95% CI [0.82, 0.87]) than in the task-present condition (M_{OVs} = 0.75, 95% CI [0.71, 0.78], M_{SVs} = 0.76, 95% CI [0.72, 0.80]). The interaction between the interpolated task and the probe was also significant, F (2, 76) = 4.56, p = 0.013, $\eta_p^2 = 0.11$. A simple-effects analysis showed that the performance in the task-absent condition was better than that in the task-present condition across the color ($M_{\text{task-absent}}$ = 0.93, 95% CI [0.90, 0.95] vs. $M_{\text{task-present}}$ = 0.84, 95% CI [0.80, 0.88]), F (1, 38) = 30.64, $p < 0.001$, $\eta_p^2 = 0.45$, shape ($M_{\text{task-absent}}$ = 0.88, 95%

CI [0.85, 0.90] vs. $M_{task\text{-}present}$ = 0.79, 95% CI [0.76, 0.83]), F (1, 38) = 32.19, $p < 0.001$, $\eta_p^2 = 0.46$, and binding conditions ($M_{task\text{-}absent}$ = 0.77, 95% CI [0.75, 0.80] vs. $M_{task\text{-}present}$ = 0.64, 95% CI [0.61, 0.66]), F (1, 38) = 96.96, $p < 0.001$, $\eta_p^2 = 0.72$. The interaction between the cognitive style and the probe was not significant, F (2, 76) = 1.07, $p = 0.349$, $\eta_p^2 = 0.03$.

The three-way interaction was significant, F (2, 76) = 5.63, $p = 0.005$, $\eta_p^2 = 0.13$. A simple-effects analysis showed that in the task-absent condition, the OVs performed better than the SVs in the binding condition, F (1, 38) = 14.08, $p < 0.001$, $\eta_p^2 = 0.27$, but not in the color condition, F (1, 38) = 0.987, p = 0.327, $\eta_p^2 = 0.03$, or the shape condition, F (1, 38) = 0.900, $p = 0.349$, $\eta_p^2 = 0.02$. On the other hand, in the task-present condition, the OVs and SVs performed comparably across the color, F (1, 38) = 0.345, $p = 0.561$, $\eta_p^2 = 0.01$, shape, F (1, 38) = 0.004, $p = 0.950$, $\eta_p^2 = 0.00$, and binding probe conditions, F (1, 38) = 0.96, $p = 0.335$, $\eta_p^2 = 0.03$ (see Figure A). The results revealed patterns consistent with those obtained from the combined analysis, suggesting that the main findings were not driven by a specific condition but rather reflected a general effect across stimulus types.

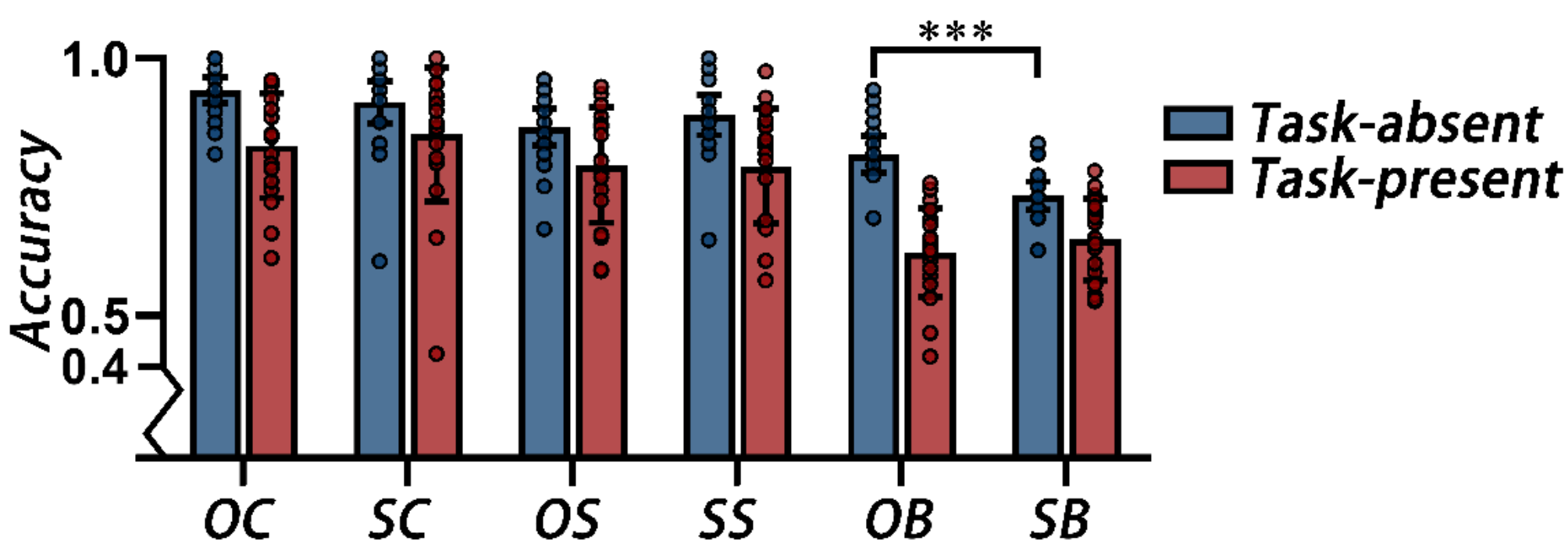

Figure A. The mean accuracy for color, shape, and binding conditions in Experiment 2; each dot corresponds to the data of an individual participant. Bars represent the 95% CIs. OC = Object visualizers' performance in the color probe condition, SC = Spatial visualizers' performance in the color probe condition, OS = Object visualizers' performance in the shape probe condition, SS = Spatial visualizers'

performance in the shape probe condition, OB = Object visualizers' performance in the binding probe condition, SB = Spatial visualizers' performance in the binding probe condition, *** $p < 0.001$.

Appendix B Interpolated Task Performance

For Experiment 2, the accuracy and reaction time (RT) data were analyzed using 2 cognitive style × 2 probe type mixed, repeated-measures ANOVAs. The cognitive style (*OVs* vs. *SVs*) was the between-subjects factor and the probe type (*feature* vs. *binding*) was the within-subjects factor. For the RT data, only trials with correct responses and RTs within three standard deviations from the mean were analyzed. For Experiment 3, the accuracy and RT data were analyzed using independent-samples *t* tests comparing OVs and SVs.

The accuracy and RT of the interpolated task in Experiments 2 and 3 are shown in Table B. In Experiment 2, the main effects of cognitive style and probe type were not significant for accuracy (p_1 = 0.621; p_2 = 0.694) or RT (p_1 = 0.509; p_2 = 0.867). Their interaction was also non-significant, both for accuracy (p = 0.220) and RT (p = 0.686). In Experiment 3, no significant difference was observed between OVs and SVs in accuracy (p = 0.744) or RT (p = 0.794). The results suggest that the interpolated task exerted a similar effect both across the cognitive styles and the probe types.

Table B. Accuracy and RT of the interpolated task in each probe condition in Experiments 2 and 3.

Exp.	Probe Type	Accuracy [95% CI]		RT (*ms*) [95% CI]	
		OVs	SVs	OVs	SVs
2	Feature	0.86 [0.83, 0.89]	0.85 [0.82, 0.88]	1019 [968, 1070]	988 [937, 1039]
	Binding	0.83 [0.78, 0.88]	0.86 [0.82, 0.91]	1015 [952, 1078]	998 [934, 1061]
3	Binding	0.84 [0.79, 0.88]	0.83 [0.78, 0.88]	1049 [988, 1109]	1037 [975, 1100]

* OVs = Object visualizers, SVs = Spatial visualizers, Exp. = Experiment.

Appendix C Additional Analyses of Sensitivity and False-Alarm Rate

Appendix C.1 Experiment 2

Sensitivity (d′) and false-alarm rate were analyzed using 2 (cognitive style) × 2 (interpolated task) × 2 (probe type) mixed repeated-measures ANOVA as used for the analysis of accuracy. The cognitive style (*OVs* vs. *SVs*) was the between-subjects factor, and the interpolated task and the probe type (*feature* vs. *binding*) were the within-subjects factors.

Sensitivity. The main effect of cognitive style was not significant, $F(1, 38) = 0.20$, $p = 0.654$, $\eta_p^2 = 0.01$. Sensitivity did not differ significantly between OVs ($M = 1.74$, 95% CI [1.54, 1.94]) and SVs ($M = 1.68$, 95% CI [1.48, 1.88]). The main effect of interpolated task was significant, $F(1, 38) = 201.90$, $p < 0.001$, $\eta_p^2 = 0.84$. Sensitivity was higher in the task-absent condition ($M = 2.14$, 95% CI [1.98, 2.31]) than in the task-present condition ($M = 1.28$, 95% CI [1.13, 1.42]). The main effect of probe type was significant, $F(1, 38) = 178.58$, $p < 0.001$, $\eta_p^2 = 0.83$. Sensitivity was higher in the feature condition ($M = 2.27$, 95% CI [2.07, 2.46]) than in the binding condition ($M = 1.16$, 95% CI [1.03, 1.28]).

The interaction between cognitive style and interpolated task was significant, $F(1, 38) = 6.79$, $p = 0.013$, $\eta_p^2 = 0.15$. Simple-effects analyses showed that in the task-absent condition, the difference between OVs ($M = 2.25$, 95% CI [2.02, 2.49]) and SVs ($M = 2.03$, 95% CI [1.80, 2.26]) was not significant, $F(1, 38) = 1.88$, $p = 0.179$, $\eta_p^2 = 0.05$. In the task-present condition, the difference between OVs ($M = 1.23$, 95% CI [1.03, 1.43]) and SVs ($M = 1.33$, 95% CI [1.12, 1.53]) was not significant, $F(1, 38) = 0.46$, $p = 0.502$, $\eta_p^2 = 0.01$. The interaction between cognitive style and probe type was not significant, $F(1, 38) = 3.18$, $p = 0.082$, $\eta_p^2 = 0.08$. The interaction between interpolated task and probe

type was not significant, F (1, 38) = 0.86, p = 0.359, η_p^2 = 0.02.

The three-way interaction was significant, F (1, 38) = 7.03, p = 0.012, η_p^2 = 0.16. Simple-simple-effects analyses showed that in the feature task-absent condition, the difference between OVs (M = 2.68, 95% CI [2.35, 3.01]) and SVs (M = 2.78, 95% CI [2.45, 3.12]) was not significant, F(1, 38) = 0.21, p = 0.648, η_p^2 = 0.01. In the feature task-present condition, the difference between OVs (M = 1.77, 95% CI [1.48, 2.06]) and SVs (M = 1.83, 95% CI [1.55, 2.12]) was not significant, F(1, 38) = 0.10, p = 0.753, η_p^2 = 0.00. In the binding task-absent condition, OVs (M = 1.83, 95% CI [1.61, 2.06]) showed higher sensitivity than SVs (M = 1.28, 95% CI [1.06, 1.51]), F(1, 38) = 12.13, p = 0.001, η_p^2 = 0.24. In the binding task-present condition, the difference between OVs (M = 0.69, 95% CI [0.49, 0.89]) and SVs (M = 0.82, 95% CI [0.62, 1.02]) was not significant, F(1, 38) = 0.83, p = 0.368, η_p^2 = 0.02.

To follow up the significant three-way interaction, task costs were compared between OVs and SVs separately for feature and binding probes using independent-samples t tests. For feature probes, the cost did not differ significantly between OVs and SVs (OVs: M = 0.91, 95% CI [0.62, 1.20]; SVs: M = 0.95, 95% CI [0.66, 1.24]), t (38) = -0.21, p = 0.833, Cohen's d = -0.07. For binding probes, the cost was greater for OVs than for SVs (OVs: M = 1.14, 95% CI [0.91, 1.37]; SVs: M = 0.46, 95% CI [0.24, 0.69]), t (38) = 4.25, $p < 0.001$, Cohen's d = 1.35.

False-alarm rate. The main effect of cognitive style was not significant, F (1, 38) = 2.66, p = 0.111, η_p^2 = 0.07. The false-alarm rate did not differ significantly between OVs (M = 0.25, 95% CI [0.20, 0.30]) and SVs (M = 0.20, 95% CI [0.15, 0.24]). The main effect of interpolated task was significant, F (1, 38) = 45.60, $p < 0.001$, η_p^2 = 0.55. The false-alarm rate was higher in the task-present condition (M = 0.28, 95% CI [0.24, 0.33]) than in the task-absent condition (M = 0.17, 95% CI [0.14, 0.19]). The main effect of probe type was significant, F (1, 38) = 70.67, $p < 0.001$, η_p^2 = 0.65. The false-alarm rate was higher

in the binding condition (M = 0.29, 95% CI [0.25, 0.34]) than in the feature condition (M = 0.15, 95% CI [0.12, 0.18]). The interaction between cognitive style and interpolated task was significant, F (1, 38) = 4.18, p = 0.048, η_p^2 = 0.10. Simple-effects analyses showed the following pattern. In the task-absent condition, the difference between OVs (M = 0.17, 95% CI [0.14, 0.21]) and SVs (M = 0.16, 95% CI [0.12, 0.20]) was not significant, F (1, 38) = 0.42, p = 0.521, η_p^2 = 0.01. In the task-present condition, the difference between OVs (M = 0.33, 95% CI [0.26, 0.39]) and SVs (M = 0.24, 95% CI [0.18, 0.30]) was not significant, F (1, 38) = 3.94, p = 0.054, η_p^2 = 0.09. The interaction between cognitive style and probe type was not significant, F (1, 38) = 1.85, p = 0.182, η_p^2 = 0.05. The interaction between interpolated task and probe type was not significant, F (1, 38) = 3.97, p = 0.054, η_p^2 = 0.10. The three-way interaction was not significant, F (1, 38) = 0.69, p = 0.411, η_p^2 = 0.02.

Appendix C.2 Experiment 3

Sensitivity and false-alarm rate were analyzed using the 2 (cognitive style) × 2 (interpolated task) mixed repeated-measures ANOVA. The cognitive style was the between-subjects factor, and the interpolated task was the within-subjects factor.

Sensitivity. The main effect of cognitive style was not significant, F (1, 33) = 2.60, p = 0.116, η_p^2 = 0.07. Sensitivity did not differ significantly between OVs (M = 1.45, 95% CI [1.26, 1.65]) and SVs (M = 1.23, 95% CI [1.04, 1.43]). The main effect of interpolated task was significant, F (1, 33) = 94.35, p < 0.001, η_p^2 = 0.74. Sensitivity was higher in the task-absent condition (M = 1.71, 95% CI [1.52, 1.89]) than in the task-present condition (M = 0.98, 95% CI [0.86, 1.10]). The interaction between cognitive style and interpolated task was not significant, F (1, 33) = 1.39, p = 0.246, η_p^2 = 0.04.

False-alarm rate. The main effect of cognitive style was significant, F (1, 33) = 5.30, p = 0.028, η_p^2 = 0.14. The false-alarm rate was higher for SVs (M = 0.32, 95% CI [0.25, 0.39]) than for OVs (M =

0.20, 95% CI [0.13, 0.27]). The main effect of interpolated task was significant, $F(1, 33) = 28.72$, $p < 0.001$, $\eta_p^2 = 0.47$. The false-alarm rate was higher in the task-present condition ($M = 0.31$, 95% CI [0.26, 0.37]) than in the task-absent condition ($M = 0.21$, 95% CI [0.16, 0.26]). The interaction between cognitive style and interpolated task was not significant, $F(1, 33) = 0.49$, $p = 0.489$, $\eta_p^2 = 0.02$.

Appendix D Working Memory Retention Duration in Experiment 2

Working memory retention duration in Experiment 2 was analyzed using the 2 (cognitive style) × 2 (interpolated task) × 2 (probe type) mixed repeated-measures design. The cognitive style (*OVs* vs. *SVs*) was the between-subjects factor, and the interpolated task and the probe type (*feature* vs. *binding*) were the within-subjects factors.

The main effect of cognitive style was not significant, $F(1, 38) = 0.14$, $p = 0.708$, $\eta_p^2 = 0.00$. Maintenance time did not differ significantly between OVs ($M = 2890$ ms, 95% CI [2799, 2980]) and SVs ($M = 2913$ ms, 95% CI [2823, 3004]). The main effect of interpolated task was significant, $F(1, 38) = 671.73$, $p < 0.001$, $\eta_p^2 = 0.95$. Maintenance time was longer in the task-present condition ($M = 3538$ ms, 95% CI [3464, 3612]) than in the task-absent condition ($M = 2265$ ms, 95% CI [2178, 2352]). The main effect of probe type was not significant, $F(1, 38) = 0.85$, $p = 0.363$, $\eta_p^2 = 0.02$. Maintenance time did not differ significantly between the feature condition ($M = 2912$ ms, 95% CI [2849, 2975]) and the binding condition ($M = 2891$ ms, 95% CI [2819, 2963]). The interaction between cognitive style and interpolated task was significant, $F(1, 38) = 4.25$, $p = 0.046$, $\eta_p^2 = 0.10$. Simple-effects analyses showed that in the task-absent condition, the difference between OVs ($M = 2203$ ms, 95% CI [2079, 2326]) and SVs ($M = 2328$ ms, 95% CI [2204, 2451]) was not significant, $F(1, 38) = 2.11$, $p = 0.154$, $\eta_p^2 = 0.05$. In the task-present condition, the difference between OVs ($M = 3577$ ms, 95% CI [3472, 3682]) and SVs ($M = 3499$ ms, 95% CI [3394, 3604]) was not significant, $F(1, 38) = 1.12$, $p = 0.297$, $\eta_p^2 = 0.03$. The

interaction between cognitive style and probe type was not significant, F (1, 38) = 1.35, p = 0.253, η_p^2 = 0.03. The interaction between interpolated task and probe type was not significant, F (1, 38) = 0.32, p = 0.574, η_p^2 = 0.01. The three-way interaction was not significant, F (1, 38) = 0.82, p = 0.370, η_p^2 = 0.02.

The significant Cognitive Style × Interpolated Task interaction indicated that the increase in retention duration from the task-absent to the task-present condition was greater for OVs than for SVs, although the simple effects comparing OVs and SVs within each task condition were not significant. However, the nonsignificant three-way interaction indicated that this difference was not specific to binding relative to feature maintenance and therefore did not parallel the critical three-way interaction observed in memory performance.
